# Nature-derived microneedles with metal-polyphenolic networks encapsulation for chronic soft tissue defects repair: Responding and remodeling the regenerative microenvironment

**DOI:** 10.1016/j.mtbio.2025.101539

**Published:** 2025-02-01

**Authors:** Chengyang Zhu, Zun Fan, Zhijie Cheng, Jun Yin, Lei Qin, Xin Zhao

**Affiliations:** Department of General Surgery, The First Affiliated Hospital of Soochow University, Suzhou, 215006, PR China

**Keywords:** Microneedles, Metal-polyphenolic networks, Drug delivery, Chronic soft tissue defects, Regenerative microenvironment

## Abstract

The treatment outcomes of traditional patches for chronic soft tissue defects (CSTDs) are unsatisfactory in clinical, owing to the lack of intrinsic bioactivities to orchestrate the intricate regenerative process. To tackle this deficiency, nature-derived microneedles (NMs) composed of silk methacrylate and snail mucus are developed in this study. The resultant NMs have excellent mechanical strength and biological adhesiveness, ensuring suture-free but reliable fixation on implanted site. To enhance the intrinsic bioactivities, metal-polyphenolic networks (MPNs) coordinated from copper (Cu) and curcumin (Cur) are designed and encapsulated into NMs. Cu-Cur MPNs harness the anti-oxidative and anti-inflammatory properties of Cur with the pro-angiogenic properties of Cu, targeting different negative aspects in CSTDs repair. Furthermore, the pH-responsive disassembly of Cu-Cur MPNs can respond to the acidic microenvironment, allowing for burst-free and on-demand drug delivery. Both in-vitro and in-vivo experiments demonstrate that NMs with Cu-Cur MPNs encapsulation (Cu-Cur-NMs) can restore redox homeostasis, reduce inflammatory response, and promote blood vessel formation, thus remodeling the regenerative microenvironment to greatly improve the repair quality of CSTDs. Therefore, the combined advantages of microneedles-based patch system and MPNs-based nanotherapeutic agent are explored for the first time, and our proposed Cu-Cur-NMs represent a multifunctional and promising device for CSTDs repair.

## Highlights

• Cu-Cur-NMs integrate advantages of microneedles and metal-polyphenolic networks.
• Cu-Cur-NMs realize reliable fixation on implanted site without need of sutures.
• Cu-Cur-NMs control drug delivery in responsive to regenerative microenvironment.
• Cu-Cur-NMs improve repair quality by remodeling regenerative microenvironment.

## Graphical abstract

**Figure:**
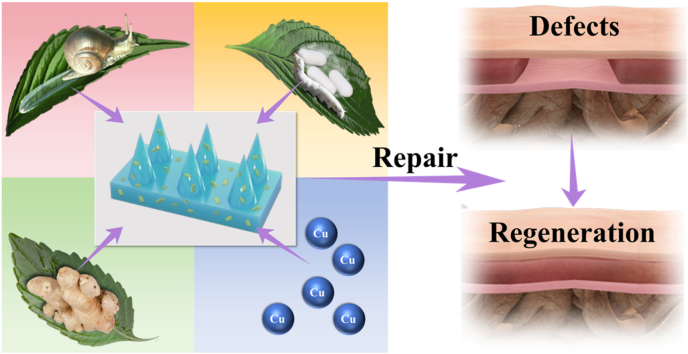

## 1 Introduction

Soft tissue defects caused by trauma, burns, and diseases remain a pressing public health issue because of their widespread morbidity and huge economic burdens [1,2]. More seriously, various pathologic conditions, such as vascular dysfunction, resistant infection, diabetic or other metabolic disorders, can disturb the normal process of tissue repair, resulting in the formation of chronic soft tissue defects (CSTDs) [3,4]. Increasing evidences have confirmed that CSTDs are often trapped in the augmented oxidative stress and prolonged inflammatory response, so that they fail to progress toward complete regeneration [5,6]. Upon injury, neutrophils, macrophages, and other immune cells are quickly recruited to conduct debridement of necrotic tissues [7]. However, the activated state of these immune cells is not well-controlled because of the immunomodulatory dysfunction associated with above pathologic conditions, leading to the excessive production of reactive oxygen species (ROS) and inflammatory cytokines in defected site [8]. Consequently, the imbalance in local microenvironment impedes repair process by restraining the function of regeneration-related cells, formation of new blood vessels, and deposition of extracellular matrix (ECM), leading to the structural and functional deficiencies in CSTDs [9,10]. Traditionally, the implantation of surgical patches including synthetic ones from macromolecular polymers and biological ones from mammalian collagen-rich tissues is recommended to restore the integrity of defected tissues by providing robust mechanical support [11,12]. However, the effectiveness of their practical applications is severely limited, owing to the lack of intrinsic bioactivities to target the varied negative factors and remodel the harsh regenerative microenvironment [13]. Therefore, there is an urgent need to develop more advanced strategies to orchestrate the intricate repair process of CSTDs [14].

In past decades, emerging works in exploration of microneedles have enabled the design of a costless, effective, and smart patch system for biomedical applications [15,16]. The microneedles consisting of miniaturized projections can perforate deep into implanted site, and deliver bioactive cargos locally in a minimally invasive manner [[17], [18], [19]]. Furthermore, the utilization of this patch system also eliminates the need for suture fixation that may cause secondary damage to necrotic tissues and foreign body reaction during repair process [20]. Therefore, various synthetic, semi-synthetic, and natural polymers are employed to fabricate a large library of microneedles, which show great advantages in skin, cardiac, corneal, vascular, and bone repair [21,22]. In this study, to meet the requirements for CSTDs repair, silk methacrylate (SilMA) and snail mucus (SM) are chosen to develop a novel kind of nature-derived microneedles (NMs). Silk fibroin is one of the most widely used natural biomaterials for tissue engineering, because of its excellent biocompatibility and mechanical strength [23]. After being modified with methacrylate group, SilMA can be photo-crosslinked to fabricate predesigned constructs under physiological conditions [24]. Meanwhile, our previous study has reported that SM is another promising natural product with biological adhesiveness to wet tissues [25]. In addition, the abundant polysaccharides and glycoproteins in SM have potential pharmacological activities, such as regulation of host cell migration, blood vessel formation, and chronic inflammatory response [[26], [27], [28]]. Although either SilMA or SM has been demonstrated in promoting tissue repair, the combined utilization of them to design NMs for the management of CSTDs remains unexplored to the best of our knowledge.

Limited efficiency of the conventional drugs in clinical practice has provoked the development of versatile nanotherapeutics derived from bioactive molecules [29]. The flexibility to design nanoscale agents including composition, size, morphology, stimuli responsiveness, and pharmacodynamic behavior, makes nanotherapeutics a unique platform for multimodal synergistic therapy, which provides a breakthrough for the treatment of various diseases [30,31]. Among them, the key role of metal-polyphenolic networks (MPNs), a distinctive supramolecular nanostructure, is highlighted in bridging nanotechnology and basic pharmacology [32]. Through matching the therapeutic effects imparted by metal ions and polyphenolic ligands, MPNs have shown multiple and diverse functions in promoting tissue repair [33]. In addition, the coordination bonds of MPNs can break up in a responsive manner to the acidic microenvironment created by persistent inflammatory response, enabling burst-free and on-demand delivery of both metal ions and polyphenolic ligands [34]. Therefore, a novel kind of MPNs formed by coordination assembly between copper (Cu) and curcumin (Cur) is designed in this study, and encapsulated into the matrix of NMs to further improve the intrinsic bioactivities of resultant patch system. Cu is the third most abundant trace element in human body, and plays essential roles in various life activities [35,36]. Strikingly, because of the superior pro-angiogenic and anti-bacterial properties, Cu-based nanotherapeutics have also attracted considerable attentions in the regenerative field [37]. As a common kind of polyphenols extracted from traditional Chinese herb turmeric, Cur has made notable contributions in ancient and modern medicine, attributed to its excellent anti-oxidative and anti-inflammatory properties [38]. However, low aqueous solubility, poor tissue absorption, and rapid metabolism make Cur unsuitable for local administration in CSTDs [39]. Fortunately, after transforming Cur molecules into Cu-Cur MPNs, these deficiencies can be resolved by taking physicochemical advantages of nanoscale agents [40].

**Fig. 1:**
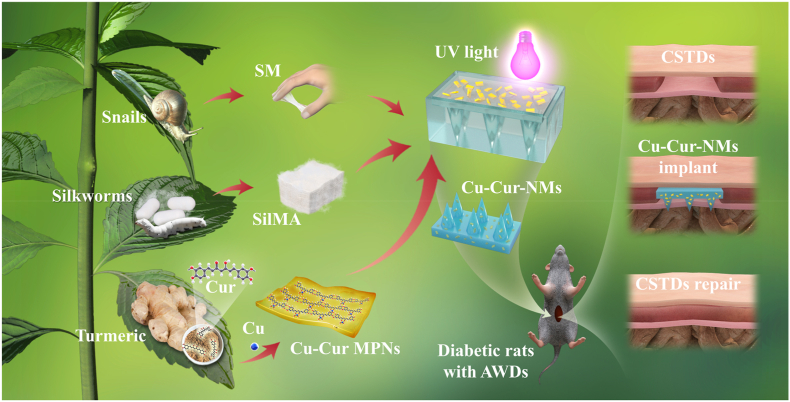
Schematic illustrations of Cu-Cur-NMs fabricated with Cu-Cur MPNs embedded into NMs and their application in diabetic rats with AWDs. The NMs were derived from ultraviolet (UV) curing of SilMA and SM, while the MPNs were derived from coordination assembly of Cu and Cur. All the synthetic components of Cu-Cur-NMs were natural building blocks, and could leverage the unique properties of Cu-Cur MPNs and NMs-based delivery system to dramatically improve the repair quality of CSTDs.

Herein, NMs with Cu-Cur MPNs encapsulation (Cu-Cur-NMs) are proposed for the first time (Fig. 1). Considering the scaffold support and drug delivery performance of NMs, along with the multiple bioactivities and microenvironmental responsiveness of MPNs, Cu-Cur-NMs are employed for the management of CSTDs. The microneedles composed of nature-derived SilMA and SM can replicate the chemical components, architecture, and mechanical properties of native ECM, thus providing an ideal scaffold for tissue regeneration. Furthermore, the miniaturized projections and biological adhesiveness of NMs can achieve suture-free but reliable fixation on implanted site even in the case of animal movements. On the other hand, the microchannels formed after NMs being implanted will allow deep delivery of loaded Cu-Cur MPNs to necrotic tissues, and make full use of their therapeutic effects. Using in-vitro cell experiments, it is found that Cu-Cur-NMs can improve the survival rate of C2C12 myoblasts exposed to hydrogen peroxide (H_2_O_2_) by inhibiting the intracellular level of oxidative stress. And by blocking the pathway associated with nucleotide-binding oligomerization domain-like receptor protein 3 (NLRP3) inflammasome activation, Cu-Cur-NMs can induce the macrophages polarization to M2 anti-inflammatory phenotype, while prevent their polarization to M1 pro-inflammatory phenotype. By upregulating the hypoxia-inducible factor-1α (HIF-1α)/vascular endothelial growth factor (VEGF) signaling, Cu-Cur-NMs can also promote the migration, proliferation, and tube formation of human umbilical vein endothelial cells (HUVECs). Using diabetic rat models of abdominal tissue defects (AWDs), it is found that Cu-Cur-NMs can remodel the regenerative microenvironment by restoring redox homeostasis, reducing inflammatory response, and promoting blood vessel formation, which significantly improve the repair quality of CSTDs. Collectively, our proposed Cu-Cur-NMs appear as a promising multifunctional patch system and hold great potential for clinical translation.

## 2 Materials and methods

### 2.1 Materials

Copper sulfate (CuSO_4_), Cur, sodium hydroxide (NaOH), and polyvinyl pyrrolidone (PVP) were purchased from Sigma-Aldrich (St. Louis, MO, USA). SilMA and lithium phenyl (2,4,6-trimethylbenzoyl) phosphinate (LAP) were purchased from Engineering For Life (Suzhou, China). 1,1-Diphenyl-2-picryl-hydrazyl (DPPH) and 2,2′-azino-bis (3-ethylbenzothiazoline-6-sulfonic acid) (ABTS) free radical scavenging capacity assay kits were purchased from Solarbio Science & Technology (Beijing, China). Methylthiazolyldiphenyl-tetrazolium bromide (MTT) assay kit and 2′,7′-dichlorodihydrofluorescein diacetate (DCFH-DA) fluorescent probe were purchased from AbMole Bioscience (Houston, TX, USA). Live/dead (Calcein-AM/PI) cytotoxicity and Cell Counting Kit-8 (CCK-8) assay kits were purchased from KeyGEN Biotechnology (Nanjing, China). Hoechst 33342, 4′,6-diamidino-2-phenylindole (DAPI), phosphate buffered saline (PBS), and rhodamine phalloidin were purchased from Yeasen Biotechnology (Shanghai, China). Lipopolysaccharide (LPS), adenosine triphosphate (ATP), and streptozotocin (STZ) were purchased from MedChemExpress (Monmouth Junction, NJ, USA). High glucose Dulbecco's modified Eagle's medium (DMEM) and fetal bovine serum (FBS) were purchased from Gibco (Waltham, MA, USA). Endothelial cell basal medium-2 (EBM-2) and BulletKit were purchased from Lonza Biotechnology (Basel, Switzerland). Growth factor-reduced Matrigel was purchased from Corning Incorporated (Bedford, MA, USA). Enzyme-linked immunosorbent assay (ELISA) kits for tumor necrosis factor (TNF)-α, interleukin (IL)-6, and IL-10 were purchased from Signalway Antibody (Greenbelt, MD, USA). Primary antibodies for inducible nitric oxide synthase (iNOS), CD206, caspase-1, IL-1β, HIF-1α, VEGF, 3-Nitrotyrosine, and α-smooth muscle actin (α-SMA) were purchased from Abcam (Waltham, MA, USA). Phycoerythrin (PE)-conjugated iNOS and fluorescein isothiocyanate (FITC)-conjugated CD206 antibodies were purchased from BioLegend (San Diego, CA, USA). Deionized water was used in all experiments. All other chemical reagents were of the best grade available and used as received.

### 2.2 Preparation of Cu-Cur MPNs and Cu-Cur-NMs

Firstly, Cu-Cur MPNs were synthesized via one-step coordination. In detail, 0.5 mg Cur and 4 mg CuSO_4_ were dissolved in 4 mL deionized water and stirred for 30 s, then 40 μL of NaOH solution (40 mg/mL) was gradually added until the orange mixture turned brown. Sequentially, 100 μL of PVP solution (200 mg/mL) was added, and after reaction for 15 s, Cu-Cur MPNs were collected through centrifugation (10000 rpm, 5 min) and washed 3 times with deionized water for subsequent use. Achatina fulica (white jade snail, 20–25 g) was purchased from Canguliang Technology Co. (Hangzhou, China). The live snails were sterilized in ozone atmosphere for 50 min after being cleaned with deionized water. By scratching the snail foot with a pipette tip to stimulate secretion, SM was collected. For subsequent use, the collected SM was centrifuged at 4000 rpm for 5 min, sterilized with a 0.22 μm polyvinylidene fluoride (PVDF) filter, and lyophilized into powder. 250 mg/mL SilMA, 7 mg/mL SM, and 8 mg/mL Cu-Cur MPNs were mixed in deionized water containing 5 mg/mL LAP, loaded into a polydimethylsiloxane (PDMS) template with ordered cavities, vacuumed under negative pressure for 5 min, and solidified through UV irradiation for 30 s. Finally, the resultant Cu-Cur-NMs were obtained by demolding, after being oven-dried at 40 °C for 1 h.

### 2.3 Characterization of Cu-Cur MPNs and Cu-Cur-NMs

The nanostructure and elemental distribution of Cu-Cur MPNs were observed by transmission electron microscopy (TEM, Talos F200X, Thermo Scientific) equipped with a high-angle annular dark-field (HAADF) detector and an energy dispersive spectrometry (EDS) mapping. The chemical composition and bonding structure of CuSO_4_, Cur, and Cu-Cur MPNs were determined by an X-ray photoelectron spectroscopy (XPS, K-Alpha, Thermo Scientific) and a spectrophotometer (Genesys150, Thermo Scientific). The thermogravimetric (TG) analysis of Cur and Cu-Cur MPNs was performed using a TG analyzer (TGA209F1, Netzsch), with the samples heated from 30 to 800 °C under an N_2_ atmosphere. Then, the overall and local morphology of Cu-Cur-NMs were observed by a stereomicroscopy (JSZ6S, Jiangnan novel optics) and fluorescent microscopy (EVOS M7000, Thermo Scientific). After spraying gold, the detailed microstructure of Cu-Cur-NMs was explored by using scanning electron microscopy (SEM, Sigma300, ZEISS).

### 2.4 Anti-oxidative activities and pH-responsive disassembly of Cu-Cur MPNs

The anti-oxidative activities of Cu-Cur MPNs were evaluated by determining their capacities to scavenge DPPH free radicals. Briefly, 100 μL of deionized water (control) or test samples were mixed with 4 mL DPPH working solution (40 μg/mL) for 2 min. Then, optical absorbance (OD) value of the mixture was determined at 517 nm wavelength using a microplate reader (Epoch EXL800, BioTek). The DPPH scavenging capacity was obtained using the following equation: DPPH scavenge (%) = (A_0_ - A_s_)/A_0_ × 100 %, in which A_0_ and A_s_ were the OD values of control and test samples, respectively. The trolox-equivalent anti-oxidative capacity (TEAC) values of Cu-Cur MPNs were measured strictly in accordance with the instructions of ABTS assay kit. Briefly, 250 μL of test samples were mixed with 4 mL ABTS working solution. After 2 min incubation, the resultant mixture was measured at 734 nm wavelength by a microplate reader. The TEAC value was obtained using the following equation based on a standard curve: TEAC (mM) = 1.6985 - 1.0556 × A_s_, in which A_s_ was the OD value of test samples. Each sample was repeated 3 times.

To evaluate the pH-responsive disassembly of Cu-Cur MPNs, a certain amount was immersed into 3 mL buffer solution with different pH values (5.0, 6.0, 7.0, and 7.4). At predetermined timepoints, 300 μL supernatant was collected after centrifugation (10000 rpm, 5 min) and refreshed with the same volume of buffer solution. The content of released Cur in collected supernatant was measured at 420 nm wavelength by a microplate reader. Meanwhile, the content of released Cu in collected supernatant was measured via an inductively coupled plasma optical emission spectrometer (Avio200, PerkinElmer). Each sample was repeated 3 times.

### 2.5 Mechanical strength, penetration ability, adhesive force, drug release, swelling and degradation properties of Cu-Cur-NMs

The stress-strain curves of Cu-Cur-NMs with different concentrations of SilMA were recorded via a tensile testing machine (CMT 6103, MTS/SANS), using porcine acellular dermal matrix (PADM) as control. The samples with a length of 30 mm and width of 10 mm were stretched at a speed of 10 mm/min, and the test continued until the samples were broken. To calculate the penetration number (%), Cu-Cur-NMs with different concentrations of SilMA were applied to the rat abdominal wall, and the number of left microchannels was counted. By dividing the number of left microchannels in abdominal wall by the number of microneedle arrays in Cu-Cur-NMs, the penetration number (%) was obtained. To calculate the penetration depth (%), Cu-Cur-NMs with different concentrations of SilMA were pressed to the agarose block, and the depth of left microchannels was measured. By dividing the depth of left microchannels in agarose block by the height of microneedle arrays in Cu-Cur-NMs, the penetration depth (%) was obtained. The adhesive force of Cu-Cur-NMs with different concentrations of SM was calculated by a self-made device (Fig. S1). Pigskin was fixed on the center of lifting platform, while Cu-Cur-NMs were fixed on the lower end of small column. After applying adequate weight to the samples for 30 s to ensure a close contact, deionized water was gradually added into the beaker until Cu-Cur-NMs and pigskin were separated. The adhesive force was calculated as follows: F = 0.98 × m/s, where m was the mass of water and s was the contact area [41]. Each sample was repeated 3 times.

For drug release test, the prepared Cu-Cur-NMs were immersed into 5 mL buffer solution with different pH values (5.0, 6.0, 7.0, and 7.4). At predetermined timepoints, the contents of released Cur and Cu in collected supernatant were measured as described before. For swelling test, the prepared Cu-Cur-NMs were initially weighed as W_0_, and then immersed into excess PBS solution at 37 °C. At predetermined timepoints, the samples were taken out, dried by blotting on filter paper, and re-weighed as W_t_. The swelling ratio was calculated using the following equation: Swelling ratio (%) = (W_t_ - W_0_)/W_0_ × 100 %. For degradation test, the prepared Cu-Cur-NMs were equilibrated in excess PBS solution for 24 h, before the initial masses were recorded as W_0_. Then, the samples were incubated in excess PBS solution at 37 °C, and the remaining masses were recorded as W_t_ at predetermined timepoints. The degradation rate was calculated using the following equation: Degradation rate (%) = (W_0_ - W_t_)/W_0_ × 100 %. Each sample was repeated 3 times.

### 2.6 Cell experiments related to C2C12 myoblasts

C2C12 myoblasts were cultured in DMEM supplemented with 10 % FBS and 1 % penicillin-streptomycin. To evaluate the cytotoxicity of Cu-Cur-NMs, C2C12 myoblasts were seeded into a 24-well plate at a density of 2 × 10^5^ cells per well, and incubated with the microneedles containing 0, 2, 4, 8, 16 mg/mL Cu-Cur MPNs for 24 h. The untreated C2C12 myoblasts were regarded as control, and MTT assay was performed to calculate the cell viability (%) in different groups following the manufacturer's instructions. To investigate the protective effects of Cu-Cur-NMs on C2C12 myoblasts in an oxidative microenvironment, these cells were seeded in a 6-well plate at a density of 3 × 10^5^ cells per well, and divided into 6 groups. For control group, C2C12 myoblasts were cultured without any treatments. While for other groups, C2C12 myoblasts were incubated with PBS (H_2_O_2_ group), blank NMs (NMs group), Cu-loaded NMs (Cu-NMs group), Cur-loaded NMs (Cur-NMs group), and Cu-Cur MPNs-loaded NMs (Cu-Cur-NMs group) for 12 h, followed by 10 μL H_2_O_2_ (3 % v/v) exposure in culture medium for another 6 h. For live/dead cell staining, the culture medium from different groups was removed and 1 mL assay buffer containing 1 μL Calcein-AM and 3 μL PI was added into each well. After being incubated for 1 h at 37 °C, the samples were photographed with a fluorescent microscopy. On the other hand, the cell-permeable DCFH-DA fluorescent probe was used to detect the ROS level in C2C12 myoblasts from above groups. After being washed with PBS for 3 times, 1 mL DMEM containing 1 μL DCFH-DA was added into each well and incubated for 30 min at 37 °C. The samples were also visualized by a fluorescent microscopy after staining the nuclei with Hoechst 33342 for 10 min. Each sample was repeated 3 times.

### 2.7 Cell experiments related to Raw264.7 macrophages

Raw264.7 macrophages were cultured in DMEM supplemented with 10 % FBS and 1 % penicillin-streptomycin. To evaluate the cytotoxicity of Cu-Cur-NMs, Raw264.7 macrophages were grouped and incubated with microneedles containing different concentrations of Cu-Cur MPNs, and the cell viability (%) was calculated using MTT assay as described before. To investigate the induced effects of Cu-Cur-NMs on Raw264.7 macrophages in an inflammatory microenvironment, these cells were seeded in a 6-well plate at a density of 3 × 10^5^ cells per well, and divided into 6 groups. For control group, Raw264.7 macrophages were cultured without any treatments. While for other groups, Raw264.7 macrophages were incubated with PBS (LPS group), NMs, Cu-NMs, Cur-NMs, and Cu-Cur-NMs for 12 h, followed by 400 ng/mL LPS exposure in culture medium for another 12 h. For immunofluorescent staining, Raw264.7 macrophages from different groups were fixed with paraformaldehyde (PFA) and permeabilized with Triton X-100. Then, primary antibodies against iNOS (1:500) and CD206 (1:500) were added and incubated overnight at 4 °C, respectively. After being washed with PBS, the secondary antibodies (1:1000) were added and incubated at room temperature for 1 h. Finally, the samples were stained with DAPI for 10 min at room temperature, and observed via a fluorescent microscopy. For flow cytometry analysis, Raw264.7 macrophages from above groups were collected and washed with PBS. These cells were stained with PE-conjugated iNOS antibody (1:200), then processed with fixation/permeabilization kit before being stained with FITC-conjugated CD206 antibody (1:200). The detection of M1 and M2 phenotypes in Raw264.7 macrophages from different groups was performed using a flow cytometer (BD FACSCalibur, BD Biosciences), and analyzed using FlowJo software.

The expression levels of inflammation-related cytokines in Raw264.7 macrophages from above groups were measured. After culture medium were harvested, ELISA kits were used to detect the productions of TNF-α, IL-6, and IL-10, according to the instructions provided by the manufacturer. To investigate the inhibitory effects of Cu-Cur-NMs on the downstream pathway of NLRP3 inflammasome activation, ASC-expressing Raw264.7 macrophages in LPS, NMs, Cu-NMs, Cur-NMs, and Cu-Cur-NMs group were additionally treated with 2.5 mg/mL ATP for 4 h. Total proteins from the above groups were extracted, subjected to SDS-PAGE electrophoresis, and transferred to PVDF membranes. Then, the membranes were incubated overnight at 4 °C with primary antibodies against IL-1β (1:1000), caspase-1 (1:1000), and β-actin (1:1000). After being washed for 3 times, the membranes were incubated with HRP-conjugated secondary antibodies (1:1000). Finally, the western blotting (WB) results were visualized by a multifunctional imaging system (Shenhua Science Technology) and the signal intensities were quantified using Image J software. Each sample was repeated 3 times.

### 2.8 Cell experiments related to HUVECs

HUVECs were cultured in EBM-2 supplemented with 1 % BulletKit and 1 % penicillin-streptomycin. To evaluate the cytotoxicity of Cu-Cur-NMs, HUVECs were grouped and incubated with microneedles containing different concentrations of Cu-Cur MPNs, and the cell viability (%) was calculated using MTT assay as described before. To investigate the positive effects of Cu-Cur-NMs on the migration, proliferation, and tube formation of HUVECs, these cells were divided into 5 groups, and incubated with PBS (control), NMs, Cu-NMs, Cur-NMs, and Cu-Cur-NMs, respectively. For migration test, 5 × 10^5^ HUVECs were suspended in 2.5 mL EBM-2 and seeded in each well of a 6-well plate. After 24 h culture, HUVECs were scratched by a pipette tip, and washed with PBS for 3 times. After being cultured for another 48 h, representative images of HUVECs from above groups were taken using an inverted microscopy. The migration rate was calculated as follows: Migration rate (%) = (A_0_ - A_48_)/A_0_ × 100 %, where A_0_ represented the initial scratch area and A_48_ represented the final scratch area after being incubated for 48 h. For proliferation test, HUVECs were seeded into a 24-well plate at a density of 2 × 10^5^ cells per well. After being cocultured with each sample for 24 h, CCK-8 assay was used to determine the proliferation rate of HUVECs from different groups. Briefly, 200 μL CCK-8 solution was added into each well, placed in an incubator for 2 h, and the OD value was measured at a wavelength of 450 nm.

For tube formation test, 250 μL Matrigel was spread onto each well of a 24-well plate and placed at 37 °C for 30 min to solidify. HUVECs were seeded at a density of 2 × 10^5^ cells per well, and cocultured with each sample for 4 h. After being stained with Calcein-AM, the representative images of HUVECs from different groups were taken by a fluorescent microscopy. The total tube number and length were quantified using Image J software, and each sample was repeated 3 times. For actin filaments staining, 5 × 10^5^ HUVECs were seeded in each well of a 6-well plate. After being cocultured with each sample for 24 h, HUVECs were fixed with PFA and permeabilized with Triton X-100. Then, rhodamine phalloidin (1:1000) was used to stain the actin filaments of HUVECs at room temperature for 1 h, followed by nuclei staining with Hoechst 33342 for 10 min. To investigate the positive effects of Cu-Cur-NMs on the activation of HIF-1α/VEGF signaling in HUVECs, WB was performed as described above, while the primary antibodies against HIF-1α (1:1000), VEGF (1:1000), and GAPDH (1:1000) were used.

### 2.9 Animal experiments

Male Sprague-Dawley rats aged 8 weeks (about 250 g) were obtained from the Experimental Animal Center of Soochow University (Suzhou, China). All animal experiments were performed under relevant protocols approved by the Animal Care Committee of Soochow University (No. 202304A0689) and conducted in compliance with regulations for the Administration of Affairs Concerning Experimental Animals of China. Diabetic conditions were created by intraperitoneal injection of 40 mg/kg STZ. After one week, anesthesia was induced by intraperitoneal injection with 50 mg/kg pentobarbital sodium, and 15 × 15 mm partial-thickness AWDs were created in diabetic rats by pair-wisely excising the external and internal oblique muscles of abdominal wall. For CSTDs treatment, these rats were divided into 4 groups (n = 3 for each group): (1) PADM group, CSTDs were repaired by PADM with Prolene sutures at each of the 4 corners; (2) Cu-Cur-PADM group, 8 mg Cu-Cur MPNs were dispersed into 1 mL PBS solution, and dropped on the surface of lyophilized PADM to fabricate Cu-Cur MPNs-loaded PADM (Cu-Cur-PADM), CSTDs were repaired by Cu-Cur-PADM with Prolene sutures at each of the 4 corners; (3) NMs group, CSTDs were repaired by NMs directly pressed into the abdominal wall; (4) Cu-Cur-NMs group, CSTDs were repaired by Cu-Cur-NMs directly pressed into the abdominal wall. Specially, the maximum power exerted by thumb was adequate to press nearly all tips of microneedles into the abdominal wall, while did not cause any damage to the structure of microneedles.

After treatment for 4 weeks, these rats were sacrificed, and the repair situation from different groups was photographed by a digital camera. The patch shrinkage rate was calculated as follows: Patch shrinkage (%) = (A_0_ - A_4_)/A_0_ × 100 %, in which A_0_ and A_4_ represented the original size of patches upon implantation and the final size of patches after implantation for 4 weeks, respectively. Subsequently, the entire repaired abdominal wall and adjacent connective tissue were harvested for histological evaluation. The tissue samples were fixed in PFA overnight, dehydrated, embedded in paraffin, and made into serial sections of 5 μm thickness. Hematoxylin-eosin (H&E) staining was performed to evaluate the general morphology of regenerated tissues and infiltration of inflammatory cells, while Masson staining was performed to evaluate the collagen deposition. To investigate the level of oxidative stress in different groups, immunohistochemical (IHC) staining was performed with the primary antibody against 3-Nitrotyrosine at a 200-fold dilution. To evaluate the phenotypic transition of macrophages in different groups, IHC staining was performed with the primary antibodies against iNOS and CD206 at a 200-fold dilution. To observe the formation of new blood vessels, IHC staining was performed with the primary antibody against α-SMA at a 200-fold dilution. The blood vessel density was calculated according to the following equation: Vessel density (%) = blood vessel area/total tissue area × 100 %.

### 2.10 Statistical analysis

The results were reported as mean ± standard deviation (SD) for at least 3 independent experiments. Data analyses in this experiment were performed by one-way analysis of variance (ANOVA) to examine differences among 3 or more groups using GraphPad Prism software. *P* < 0.05 was regarded as a significance threshold.

## 3 Results and discussion

### 3.1 Preparation and characterization of Cu-Cur MPNs

**Fig. 2:**
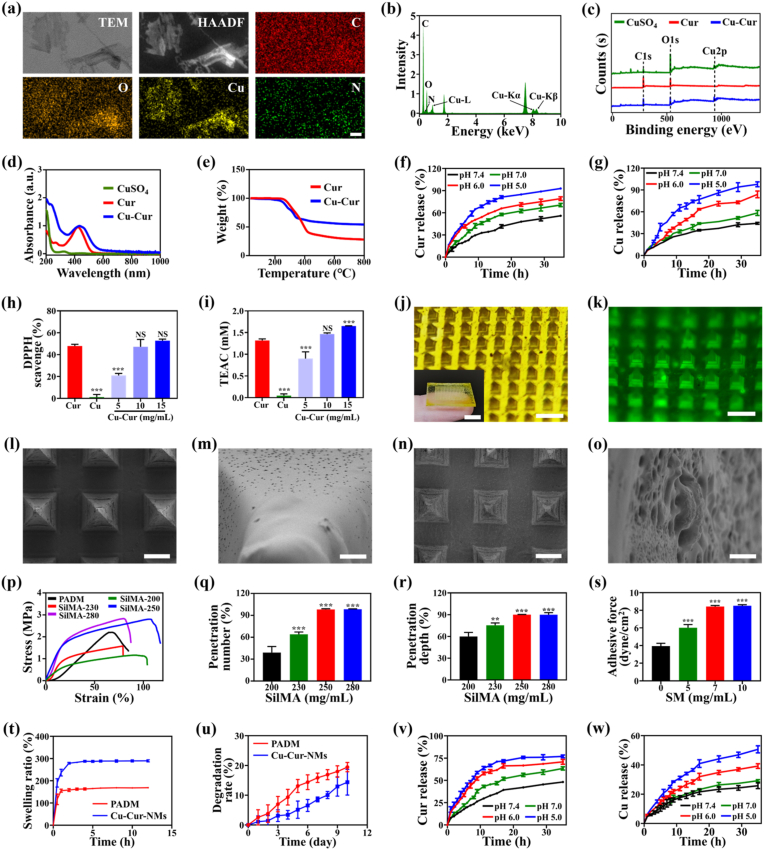
a) TEM image of Cu-Cur MPNs and corresponding HAADF image with elemental mappings of C (red), O (orange), Cu (yellow), and N (green), scale bar: 20 nm. b) EDS analysis of Cu-Cur MPNs. c) XPS analysis of CuSO_4_, Cur, and Cu-Cur MPNs. d) UV–vis adsorption spectra of CuSO_4_, Cur, and Cu-Cur MPNs. e) TG analysis of Cur and Cu-Cur MPNs. f) Cumulative release curves of Cur from Cu-Cur MPNs under different pH values. g) Cumulative release curves of Cu from Cu-Cur MPNs under different pH values. h) DPPH free radical scavenging activity of Cu-Cur MPNs. i) ABTS free radical scavenging activity of Cu-Cur MPNs. j) Optical images of intact Cu-Cur-NMs and amplified microneedle arrays, scale bar: 800 μm (5 mm in insert). k) Fluorescent image of Cu-Cur-NMs, scale bar: 700 μm. l) SEM image of blank NMs under low magnification, scale bar: 250 μm. m) SEM image of blank NMs under high magnification, scale bar: 10 μm. n) SEM image of Cu-Cur-NMs under low magnification, scale bar: 250 μm. o) SEM image of Cu-Cur-NMs under high magnification, scale bar: 10 μm. p) The stress-strain curves of Cu-Cur-NMs with different concentrations of SilMA. q) The number of microchannels penetrated on abdominal wall after implanting Cu-Cur-NMs with different concentrations of SilMA. r) The depth of microchannels penetrated on agarose block after implanting Cu-Cur-NMs with different concentrations of SilMA. s) The adhesive force of Cu-Cur-NMs on wet tissues with different concentrations of SM. t) The swelling ratio of Cu-Cur-NMs compared to PADM. u) The degradation rate of Cu-Cur-NMs compared to PADM. v) Cumulative release curves of Cur from Cu-Cur-NMs under different pH values. w) Cumulative release curves of Cu from Cu-Cur-NMs under different pH values. Error bars represented standard deviations. ∗∗*p* < 0.01, ∗∗∗*p* < 0.001, NS not significant versus first group.

In a standard experimental setup, a novel kind of MPNs was firstly synthesized through one-step coordination assembly between Cu and Cur under alkaline conditions, with the assistance of PVP as a stabilizer (Fig. S2). The resultant Cu-Cur MPNs exhibited an irregular laminar morphology, which was observed by TEM, with an ultrathin nanosheet structure (Fig. 2a). The HAADF image with elemental mappings and EDS analysis demonstrated the distributions of C, O, and Cu across the nanosheet structure (Fig. 2b). Meanwhile, the negligible distribution of N indicated that PVP was nearly removed from the surface of Cu-Cur MPNs after being washed with deionized water. Consistently, C, O, and Cu were all observed in the full-scan XPS spectra of Cu-Cur MPNs (Fig. 2c). Furthermore, one characteristic peak at 933.02 eV (Cu2p3, Cu-O) in the high resolution of Cu2p spectra and another characteristic peak at 529.74 eV (Metal-O) in the high resolution of O1s spectra, indicated the formation of Cu-phenolic hydroxyl group coordination bonds (Fig. S3). The ultraviolet–visible (UV–vis) adsorption spectra detected a characteristic peak at 434 nm of Cu-Cur MPNs similar to that of Cur, and a redshift again suggested the interaction between Cu and Cur (Fig. 2d). Finally, the TG analysis found that the mass fractions of Cu and Cur in MPNs were 36.25 % and 63.75 %, respectively, indicating that Cu and Cur coordinated in a molar ratio of approximately 3:1 (Fig. 2e). Together, these results confirmed the successful preparation of Cu-Cur MPNs, and the fabrication process could be characterized as highly efficient, environmentally safe, and economically viable.

Because the coordination bonds between metal ions and polyphenolic ligands are sensitive to acidic substances, MPNs exhibit a distinctive responsiveness to the variations of pH values [34]. Fig. 2f–g recorded the cumulative release curves of Cur and Cu after immersing Cu-Cur MPNs into the release media with different pH values. The stability of MPNs incubated at pH 7.4 guaranteed slow and sustained release kinetics, with only 55.95 % of Cur and 44.20 % of Cu released within 35 h. In contrast, the decrease of pH value in release media could promote the disassembly of coordination bonds and contribute to accelerated release kinetics. It was found that 92.84 %, 79.10 %, and 70.48 % of Cur, and 97.79 %, 83.87 %, and 58.37 % of Cu were released after 35 h incubation at pH 5.0, 6.0, and 7.0, respectively. Generally, the persistent inflammatory response in CSTDs can produce acidic substances such as lactic acid, thus lowering the pH value of local microenvironment [3]. Therefore, the pH-responsive disassembly of Cu-Cur MPNs could respond to the regenerative microenvironment of CSTDs, and realize controlled delivery of both Cur and Cu to make full use of their pharmacological effects while avoiding dose-related toxicities. Because of the active hydroxyl groups, Cur can directly interact with free radicals and is deemed as a kind of well-documented anti-oxidants [39]. However, it remained unclear whether this property was preserved after transforming Cur molecules into Cu-Cur MPNs. To address this issue, Cu-Cur MPNs were immersed into DPPH test solution, and it was found that the purple coloration of test solution faded upon the addition of Cu-Cur MPNs (Fig. S4a). Quantitative analysis revealed that Cu-Cur MPNs could scavenge DPPH free radicals in a concentration-dependent behavior (Fig. 2h). Specifically, when the concentration of Cu-Cur MPNs set as 5, 10, and 15 mg/mL, the DPPH scavenging efficiency was 20.98 %, 47.09 %, and 52.68 %, respectively. TEAC values calculated based on the ABTS assay also confirmed that higher concentrations of Cu-Cur MPNs led to more significant anti-oxidative activities (Fig. 2i and Fig. S4b). Collectively, the developed Cu-Cur MPNs preserved the anti-oxidative properties of Cur, and obtained additional pH-responsive abilities. These two biological characteristics made Cu-Cur MPNs a promising candidate for CSTDs repair, which could respond and remodel the regenerative microenvironment.

### 3.2 Preparation and characterization of Cu-Cur-NMs

Subsequently, Cu-Cur-NMs were fabricated by filling a mixed solution of SilMA, SM, and Cu-Cur MPNs into the cavities of a PDMS template under vacuum negative pressure, and photo-crosslinked through UV light irradiation (Fig. S5). After being oven-dried, the resultant Cu-Cur-NMs with a uniform 20 × 20 microneedle array were successfully detached from the negative template (Fig. 2j). Compared to blank NMs, the encapsulation of Cu-Cur MPNs did not disturb the formation of pyramid-shaped projections, with negligible alterations in both the height and diameter of microneedle arrays (Figs. S6–7). The green fluorescence observed by fluorescent microscopy indicated the even distribution of Cu-Cur MPNs in the matrix of NMs (Fig. 2k). In addition, the images from SEM showed that the encapsulation of Cu-Cur MPNs markedly improved the surface roughness and porosities of NMs (Fig. 2l–o). These morphological alterations of Cu-Cur-NMs might augment the interaction interface and frictional force with implanted site, facilitating an enhanced fixation of NMs and more efficient delivery of Cu-Cur MPNs in CSTDs.

As a patch system for restoring the integrity of CSTDs, the ideal Cu-Cur-NMs should have adequate mechanical properties to resist the exerted pressure during animal movements. The stress-strain curves of Cu-Cur-NMs with different concentrations of SilMA were recorded, and compared to commercially available PADM patches. As shown in Fig. 2p, the tensile strength and elastic modulus of PADM were 2.20 MPa and 4.29 MPa, respectively. It was found that both the tensile strength and elastic modulus of Cu-Cur-NMs were improved with the increased concentrations of SilMA. When 250 mg/mL SilMA used, the tensile strength and elastic modulus of Cu-Cur-NMs respectively reached 2.80 MPa and 10.10 MPa, both of which were slightly strengthened compared to those of PADM. As a release platform for the efficient delivery of Cu-Cur MPNs, the ideal Cu-Cur-NMs should have a solid microneedle array to create adequate microchannels on implanted site. The penetration abilities of Cu-Cur-NMs with different concentrations of SilMA were investigated by recording the number and depth of created microchannels after inserting Cu-Cur-NMs into targeted tissues (Figs. S8–9). Quantitative analysis revealed that both the number and depth of created microchannels were improved with the increased concentrations of SilMA (Fig. 2q–r). When 250 mg/mL SilMA used, the penetration number and depth on implanted site nearly reached 90 % relative to the number and height of microneedle arrays. And further increasing the concentration of SilMA to 280 mg/mL, the penetration abilities of Cu-Cur-NMs exhibited negligible improvements. Therefore, considering the mechanical properties and penetration abilities, 250 mg/mL SilMA was used to fabricate Cu-Cur-NMs in the following experiments. In addition, endowing Cu-Cur-NMs with adequate bio-adhesive properties could help them better fix and adapt to the dynamic tissues. Expectedly, the adhesive force of Cu-Cur-NMs to wet tissues was improved in a dependent manner on SM concentrations (Fig. 2s). Because further increasing the SM concentrations made no obvious sense on the bio-adhesive properties of Cu-Cur-NMs, 7 mg/mL SM was recommended in the following experiments.

The swelling behavior of implants is beneficial to accelerate repair process by absorbing excess exudates, while maintaining a moist microenvironment for tissue regeneration [42]. As shown in Fig. 2t, the swelling ration of Cu-Cur-NMs reached 289.82 % after incubation in PBS solution for 12 h, and was markedly higher than that of PADM (168.76 %), exhibiting a robust water absorption capacity. In addition, the implants used for CSTDs management should degrade at a slow rate, which can provide a long-lasting mechanical support for the defected site to prevent a recurrence [43]. Compared to that of PADM (80.55 %), Cu-Cur-NMs maintained 85.63 % of the mass after incubation in PBS solution for 10 days, verifying a satisfied degradation rate (Fig. 2u). It was worth noting that, the pH-responsive properties were preserved after encapsulating Cu-Cur MPNs into the matrix of NMs. As shown in Fig. 2v–w, the release rates of both Cur and Cu from Cu-Cur-NMs were speeded up along with the decrease of pH value in buffer solution. Specially, 76.99 %, 70.82 %, 63.50 %, and 48.19 % of Cur, and 50.71 %, 39.23 %, 29.22 %, and 25.75 % of Cu were released after 35 h incubation at pH 5.0, 6.0, 7.0, and 7.4, respectively.

### 3.3 Cu-Cur-NMs supported C2C12 myoblasts survival by alleviating oxidative stress

**Fig. 3:**
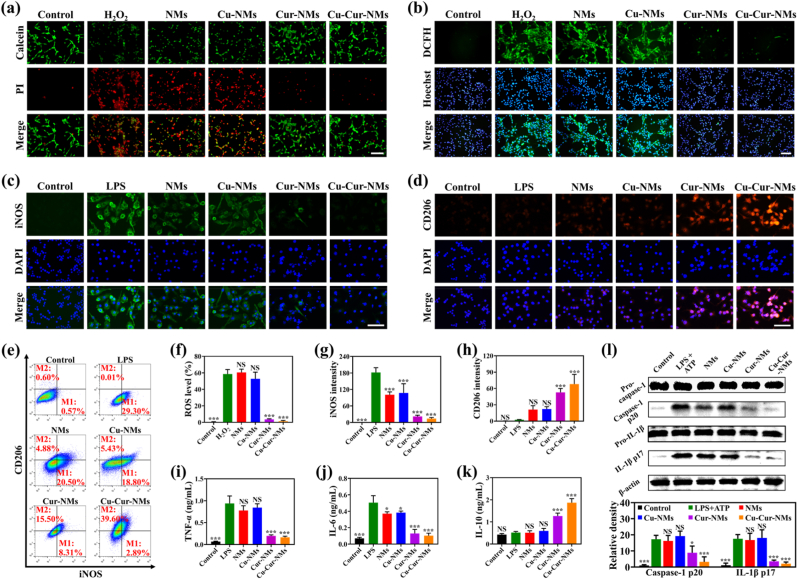
a) H_2_O_2_-exposed C2C12 myoblasts were divided into 5 groups, treated respectively with PBS (H_2_O_2_ group), NMs, Cu-NMs, Cur-NMs, and Cu-Cur-NMs, while C2C12 myoblasts not exposed to H_2_O_2_ were regarded as control group. After being stained with Calcein-AM (live cells, green) and PI (dead cells, red), fluorescent images of C2C12 myoblasts from different groups were captured, scale bar: 250 μm. b) After being stained with DCFH-DA (ROS, green) and Hoechst 33342 (cell nucleus, blue), fluorescent images of C2C12 myoblasts from different groups were captured, scale bar: 250 μm. c) LPS-exposed Raw264.7 macrophages were divided into 5 groups, treated respectively with PBS (LPS group), NMs, Cu-NMs, Cur-NMs, and Cu-Cur-NMs, while Raw264.7 macrophages not exposed to LPS were regarded as control group. After being stained with iNOS antibody (M1 phenotype, green) and DAPI (cell nucleus, blue), fluorescent images of Raw264.7 macrophages from different groups were captured, scale bar: 50 μm. d) After being stained with CD206 antibody (M2 phenotype, red) and DAPI (cell nucleus, blue), fluorescent images of Raw264.7 macrophages from different groups were captured, scale bar: 50 μm. e) Flow cytometric analysis for measuring the proportion of M1 (iNOS-labeled) and M2 (CD206-labeled) phenotype in Raw264.7 macrophages from different groups. f) Quantification of ROS level based on the images in (b). g) Quantification of iNOS intensity based on the images in (c). h) Quantification of CD206 intensity based on the images in (d). i-k) ELISA assay of TNF-α (i), IL-6 (j), and IL-10 (k) in Raw264.7 macrophages from different groups. l) WB analysis of caspase-1 and IL-1β activation in Raw264.7 macrophages from different groups and the corresponding quantitative analysis. Error bars represented standard deviations. ∗*p* < 0.05, ∗∗∗*p* < 0.001, NS not significant versus H_2_O_2_ or LPS group.

Before exploring their biological activities in cell experiments, MTT assay was performed to evaluate the cytotoxicity of Cu-Cur-NMs against C2C12 myoblasts, Raw264.7 macrophages, and HUVECs, all of which involved in the essential processes of tissue repair (Fig. S10). Not surprisingly, Cu-Cur-NMs had an excellent cytocompatibility when containing a wide range of Cu-Cur MPNs concentrations (0–8 mg/mL). As further increasing the concentration of Cu-Cur MPNs to 16 mg/mL, Cu-Cur-NMs exhibited a detectable cytotoxic effect, 8 mg/mL Cu-Cur MPNs were used to fabricate Cu-Cur-NMs in the following experiments. As well known, uncontrolled immune cells in CSTDs will generate excessive ROS to cause oxidative damage to the regeneration-related cells, leading to their death, and thus impede the repair process of CSTDs [44]. Given the anti-oxidative activities of Cu-Cur-NMs, their protective effects on C2C12 myoblasts survival in an oxidative microenvironment were measured (Fig. 3a). Specifically, compared to control group without H_2_O_2_ exposure (0.68 %), massive amounts of cell death were observed after C2C12 myoblasts exposed to H_2_O_2_ (45.73 %). The proportion of H_2_O_2_-induced dead cells failed to elicit a notable difference with the treatments of NMs (44.25 %) and Cu-NMs (46.80 %). In contrast, Cu-Cur-NMs treatment significantly reduced the death rate to 10.26 % and supported C2C12 myoblasts survival under H_2_O_2_ exposure, which slightly outperformed Cur-NMs treatment (10.97 %) (Fig. S11).

On the other hand, the intracellular levels of oxidative stress were evaluated using ROS indicator DCFH-DA fluorescent probe (Fig. 3b). Consistently, the H_2_O_2_-induced high DCFH-DA intensity in C2C12 myoblasts was alleviated by Cur-NMs and Cu-Cur-NMs treatments, while not by NMs and Cu-NMs treatments. Notably, the DCFH-DA intensity in C2C12 myoblasts treated by Cu-Cur-NMs was the lowest among all treatment groups, resembled that of control group not exposed to H_2_O_2_ (Fig. 3f). Together, these results demonstrated that Cu-Cur-NMs could effectively alleviate the intracellular level of oxidative stress in C2C12 myoblasts, and support their survival in an oxidative microenvironment. Previous studies have reported that the transformation of polyphenolic molecules into MPNs nanostructures will improve the biological activities of polyphenols by protecting their functional groups and preventing their rapid clearance [34]. Therefore, a slightly enhanced anti-oxidative activity of Cu-Cur-NMs compared to Cur-NMs was observed in these cell experiments.

### 3.4 Cu-Cur-NMs reprogrammed Raw264.7 macrophages by inhibiting NLRP3 inflammasome activation

Prolonged inflammatory condition that impairs the proliferative and remodeling phases is a distinctive pathophysiological characteristic of CSTDs, and dysregulated macrophages with excessive production of pro-inflammatory cytokines is considered as a major contributor [45]. Considering the widely-recognized anti-inflammatory activities of Cur, the effects of Cu-Cur-NMs on promoting macrophages differentiation to M2 anti-inflammatory phenotype, while preventing their differentiation to M1 pro-inflammatory phenotype were measured. After Raw264.7 macrophages exposed to LPS, the immunofluorescent signal for M1 marker (iNOS) was markedly elevated, while the immunofluorescent signal for M2 marker (CD206) remained weak compared to control group without LPS exposure (Fig. 3c–d). With the treatments of Cur-NMs and Cu-Cur-NMs, the iNOS signal in LPS-induced Raw264.7 macrophages was markedly decreased, along with an enhanced expression of CD206 signal, indicating that the differentiation to M2 phenotype was successfully realized (Fig. 3g–h). Consistently, the results from flow cytometry also confirmed that Cur-NMs and Cu-Cur-NMs treatments could effectively reprogram Raw264.7 macrophages (Fig. 3e and Fig. S12). The proportion of M2 macrophages in LPS group was only 0.01 %, which increased to 15.50 % and 39.60 % in Cur-NMs and Cu-Cur-NMs group, respectively. It was noteworthy that NMs (4.88 %) and Cu-NMs (5.43 %) treatments also had a relatively weaker effect on promoting the M2 differentiation of Raw264.7 macrophages. Previous studies have reported that the bioactive components of SM can attenuate inflammation and reprogram macrophages in diabetic wounds [26]. Therefore, the Cur in MPNs and SM in NMs might play a synergistic role in reprogramming macrophages, leading to the highest proportion of M2 macrophages observed in Cu-Cur-NMs treatment among all groups. Previous studies have reported that an array of pro-inflammatory cytokines, including TNF-α, IL-6, and IL-1β, are secreted from M1 macrophages, and instrumental in amplifying inflammatory responses to destroy the regenerative microenvironment. In contrast, an array of anti-inflammatory cytokines, such as IL-4 and IL-10, are secreted from M2 macrophages, and renowned for their immunomodulatory properties to initiate the proliferative phase of tissue repair [46,47]. Expectedly, ELISA assay in this study also demonstrated that Cu-Cur-NMs treatment significantly decreased the expressions of pro-inflammatory cytokines TNF-α and IL-6, while increased the expression of anti-inflammatory cytokine IL-10 in LPS-exposed Raw264.7 macrophages, which outperformed NMs, Cu-NMs, and Cur-NMs treatments (Fig. 3i–k).

NLRP3 inflammasome is an essential pattern recognition receptor on immune cells that can sense a large library of endogenous danger signals [48]. The aberrant activation of NLRP3 inflammasome triggers the activation of caspase-1, mediates the maturing of pro-inflammatory cytokine IL-1β, and results in uncontrolled inflammation, which underlays various chronic diseases [49]. In addition, treatment strategies that target the downstream pathway of NLRP3 inflammasome activation can promote the macrophages polarization to M2 phenotype, and hold promise to combat the chronic inflammation-driven complex disorders [50]. Therefore, it was speculated that the promoting effects of Cu-Cur-NMs treatment on reprogramming macrophages might be related to the suppression of NLRP3 inflammasome activation. To verify this hypothesis, the expression levels of pro-caspase-1, pro-IL-1β, active caspase-1 (p20), and mature IL-1β (p17) were examined by WB analysis (Fig. 3l and Fig. S13). In response to ATP and LPS exposure, the protein levels of pro-caspase-1 and pro-IL-1β in Raw264.7 macrophages remained unchanged, while the protein levels of p20 and p17 were markedly elevated compared to control group without ATP and LPS exposure, indicating that the downstream pathway of NLRP3 inflammasome was successfully activated. Although NMs and Cu-NMs treatments had no obvious alterations, Cur-NMs and Cu-Cur-NMs treatments could decrease the protein levels of p20 and p17. Obviously, the inhibitory effect of Cu-Cur-NMs treatment on NLRP3 inflammasome activation was the most significant. Collectively, these results demonstrated that Cu-Cur-NMs could prevent the aberrant activation of NLRP3 inflammasome in macrophages, promote their beneficial differentiation to M2 anti-inflammatory phenotype, reduce the production of pro-inflammatory cytokines, and enhance the production of anti-inflammatory cytokines. All these biological activities of Cu-Cur-NMs on macrophages might reshape the inflammatory microenvironment of CSTDs, and help the repair process of CSTDs step into the proliferative and remodeling phases.

### 3.5 Cu-Cur-NMs promoted angiogenic behaviors of endothelial cells by up-regulation of HIF-1α/VEGF signaling

**Fig. 4:**
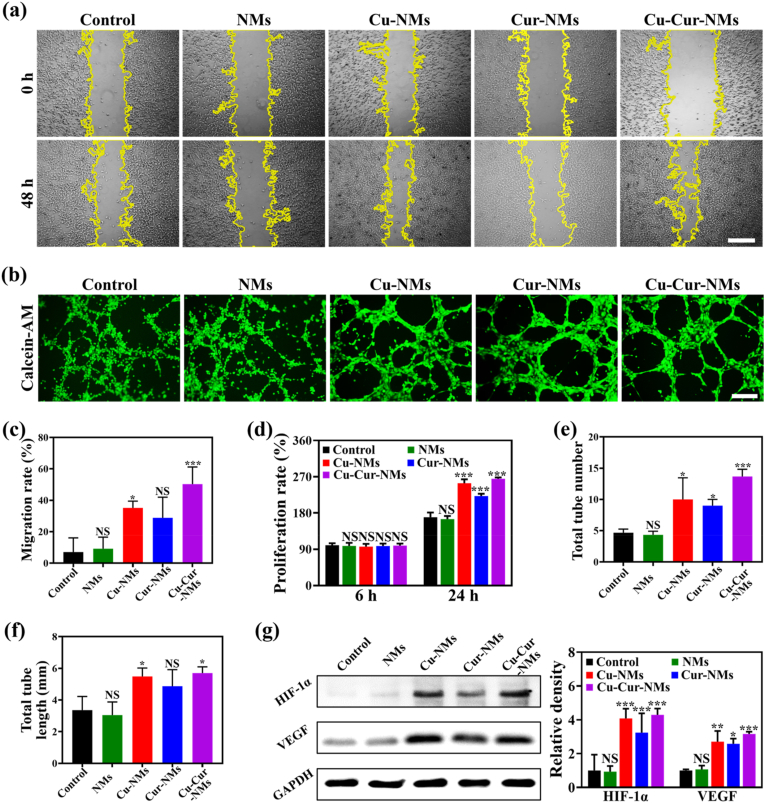
a) Migration images of HUVECs after treatment with blank control, NMs, Cu-NMs, Cur-NMs, and Cu-Cur-NMs, scale bar: 500 μm. b) Tube formation images of HUVECs from different groups, scale bar: 250 μm. c) Quantification of migration rate based on the images in (a). d) Quantification of proliferation rate based on the results of CCK-8 assay. e) Quantification of total tube number based on the images in (b). f) Quantification of total tube length based on the images in (b). g) WB analysis of HIF-1α and VEGF proteins in HUVECs from different groups and the corresponding quantitative analysis. Error bars represented standard deviations. ∗*p* < 0.05, ∗∗*p* < 0.01, ∗∗∗*p* < 0.001, NS not significant versus control group.

Delayed angiogenesis is another distinctive pathophysiological characteristic of CSTDs, that will limit the supply of nutrition and oxygen for the repair process [51]. Considering the well-documented pro-angiogenic activities of Cu, the promoting effects of Cu-Cur-NMs on the migration, proliferation, tube formation, and cytoskeleton distribution of HUVECs were measured. First, a scratch assay was employed to investigate the migration of HUVECs treated by NMs, Cu-NMs, Cur-NMs, or Cu-Cur-NMs for 48 h (Fig. 4a). Compared to control group without any treatments, Cu-NMs and Cu-Cur-NMs treatments significantly promoted the migration of HUVECs with less scratch areas remained, while NMs and Cur-NMs treatments had no obvious effects. Quantitative analysis also revealed that the migration rates of HUVECs treated by Cu-NMs and Cu-Cur-NMs were 35.16 % and 50.29 %, respectively, higher than those of blank control (7.04 %), NMs (9.19 %), and Cur-NMs (28.80 %) (Fig. 4c). Second, a CCK-8 assay was employed to investigate the 24 h proliferation of HUVECs in different groups. As shown in Fig. 4d, the proliferation rates of HUVECs treated by Cu-NMs, Cur-NMs, and Cu-Cur-NMs were 253.98 %, 221.94 %, and 264.73 %, respectively, higher than those of blank control (169.25 %) and NMs (164.73 %). Finally, Calcein-AM staining was employed to investigate the effects of above treatments on tube formation of HUVECs. As shown in Fig. 4b, HUVECs treated by Cu-NMs and Cu-Cur-NMs formed extensive and intricate tube-like structures, with the total tube number and length nearly doubled over those of control group. Unexpectedly, although HUVECs treated by NMs formed seldom tube-like structures resembled those of control group, the total tube number and length in HUVECs treated by Cur-NMs were slightly improved, indicating that Cur might also play a positive role in regulating the angiogenic behaviors of HUVECs (Fig. 4e–f). In addition, the distribution of actin filaments in HUVECs from different groups was observed after phalloidin staining. As shown in Fig. S14, Cu-Cur-NMs promoted the formation of network-like cytoskeleton in the cytoplasm of HUVECs, which outperformed the other groups. It was worth noting that, compared to Cu-NMs, an enhanced effect of Cu-Cur-NMs on promoting the migration, proliferation, tube formation, and cytoskeleton distribution of HUVECs was observed, which might be attributed to the burst-free and controlled release of Cu from Cu-Cur-NMs and the potential synergistic actions from Cur.

The hypoxic microenvironment in ischemic tissues results in an accumulation of HIF-1α, which is a major transcription factor up-regulating the expression of VEGF in the process of angiogenesis [52]. Therefore, therapeutic strategies targeted at increasing the expression of HIF-1α and its transcription activity have been proposed to restore the blood perfusion in ischemic tissues and promote the repair process. However, HIF-1α is sensitive to proteolysis and easily deactivated in CSTDs because of the enhanced effect of prolyl hydroxylases, leading to the delayed angiogenesis. Recently, Cu-based nanotherapeutics have been reported to increase the stability of HIF-1α by inhibiting prolyl hydroxylases, along with the productions of downstream pro-angiogenic growth factors [53]. To investigate whether Cu-Cur-NMs promoted the angiogenesis-associated behaviors of HUVECs by up-regulation of HIF-1α/VEGF signaling, WB analysis was performed. Compared to control group, Cu-NMs, Cur-NMs, and Cu-Cur-NMs all significantly increased the protein expressions of HIF-1α and VEGF in HUVECs, while NMs had no obvious alterations (Fig. 4g and Fig. S15). Quantitative analysis further revealed that the protein levels of both HIF-1α and VEGF were the highest in HUVECs treated by Cu-Cur-NMs. Together, these results demonstrated that Cu-Cur-NMs could activate the HIF-1α/VEGF signaling in HUVECs, and promote their migration, proliferation, tube formation, and cytoskeleton distribution. All these biological activities of Cu-Cur-NMs might restore the blood perfusion, reshape the ischemic microenvironment of CSTDs, and accelerate the repair process.

### 3.6 Cu-Cur-NMs improved the repair quality of CSTDs in vivo

**Fig. 5:**
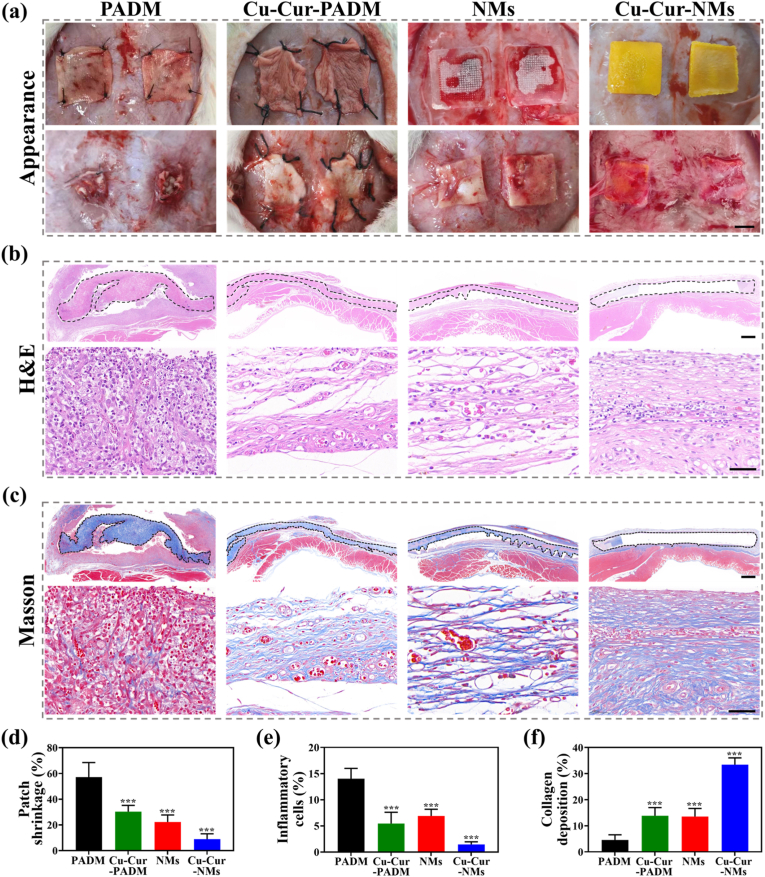
a) Bilateral AWDs in a diabetic rat model were treated by PADM, Cu-Cur-PADM, NMs, and Cu-Cur-NMs (top line), and gross observation of the repair situation after implantation for 4 weeks (bottom line), scale bar: 5 mm. b) H&E staining images from samples treated by 4 groups of patches under low (top line) and high (bottom line) magnifications, scale bars: 1 mm (top line) and 40 μm (bottom line). c) Masson staining images from samples treated by 4 groups of patches under low (top line) and high (bottom line) magnifications, scale bars: 1 mm (top line) and 40 μm (bottom line). d) Quantification of patch shrinkage based on the images in (a). e) Quantification of inflammatory cell infiltration based on the images in (b). f) Quantification of collagen deposition based on the images in (c). Error bars represented standard deviations. ∗∗∗*p* < 0.001 versus PADM group.

The in-vitro results presented above demonstrated that Cu-Cur-NMs could realize controlled delivery of bioactive components in response to the pH alteration of regenerative microenvironment. In turn, the effects of these bioactive components on scavenging free radicals, reprogramming macrophage phenotypes, and regulating HUVECs behaviors to remodel the oxidative, inflammatory, and ischemic microenvironment were confirmed, with the relative mechanism also explored. These results provided a theoretical and experimental basis for the in-vivo application of Cu-Cur-NMs for CSTDs management. In this study, bilateral 15 × 15 mm partial-thickness AWDs were created in the diabetic rats, which mimicked the clinical setting of CSTDs (Fig. S16). These defects were divided into 4 groups, PADM and Cu-Cur-PADM were fixed with Prolene sutures at each of the 4 corners, while NMs and Cu-Cur-NMs were directly pressed into the abdominal wall without sutures. In the follow-up, no evidence of abdominal bulge in the defected site was observed, indicating that the mechanical strength of both PADM and NMs was adequate to withstand the intra-abdominal pressure. After implantation for 4 weeks, the abdominal skin of diabetic rats was opened, and the repair situation from different groups was photographed. As shown in Fig. 5a, all groups of patches were remained in the implanted site despite normal animal movements, indicating that the suture-free implantation of NMs could be firmly fixed in abdominal wall, with a similar performance to Prolene sutures. In clinical, the persistent inflammation in CSTDs triggers the deformation of implanted patches, and is deemed as the major cause of chronic pain syndrome [54]. Obviously, a serious degree of deformity was exhibited in PADM, while the other groups of patches nearly remained their original shape. Quantitative measurement of the final patch area relative to its original was performed and termed as patch shrinkage. As shown in Fig. 5d, the patch shrinkage of PADM was 57.15 %, followed by Cu-Cur-PADM (30.35 %) and NMs (22.31 %), while the patch shrinkage of Cu-Cur-NMs (8.97 %) was the lowest. Therefore, the shape of Cu-Cur-NMs was not influenced by the harsh microenvironment of CSTDs, which guaranteed stable therapeutic outcomes and rare occurrence of complications. In addition, a thick layer of newly regenerated tissues wrapped around Cu-Cur-NMs, with blurred boundaries between the implanted patches and surrounding abdominal wall. However, the distributions of regenerated tissues in other groups of patches were relatively scattered, and their boundaries were still clear. Together, these macroscopic observations showed that Cu-Cur-NMs promoted the best integration with regenerated abdominal wall among all treatment groups. And given that Cu-Cur-NMs did not require suture fixation in contrast to PADM, the operation time would be shortened and iatrogenic tissue damage would be avoided in the future clinical application.

To further investigate the repair quality of AWDs in diabetic rats from different groups, histopathological examinations were performed. It was found that, all groups of patches nearly remained their original morphology after implantation in vivo for 4 weeks, consistent with the slow degradation rate observed in vitro (Fig. 5b). H&E staining images revealed an intermittent layer of rare granulation tissues distributed around the contracted patch, and large amounts of inflammatory cells accumulated in the PADM-treated samples, indicating that the persistent inflammation in local microenvironment delayed the repair process of CSTDs. In contrast, the number of infiltrated inflammatory cells was reduced, and the granulation tissues became more continuous in Cu-Cur-PADM-treated and NMs-treated samples. Strikingly, the Cu-Cur-NMs-treated samples had a completely continuous layer of abundant granulation tissues, and a minimal infiltration of inflammatory cells among all groups (Fig. 5e). On the other hand, Masson staining images revealed that the collagen fibrils formed in PADM-treated samples were scarce and irregular (Fig. 5c). Although Cu-Cur-PADM and NMs treatments could promote the collagen deposition, the most densely packed and oriented collagen fibrils were found in Cu-Cur-NMs-treated samples (Fig. 5f). Collectively, both the Cu-Cur MPNs and NMs had a positive role in the modulation of inflammatory response, formation of granulation tissues, and deposition of collagen fibrils. After a subtle combination of Cu-Cur MPNs and NMs into one patch system, the resultant Cu-Cur-NMs could further improve the repair quality of CSTDs.

### 3.7 Cu-Cur-NMs remodeled the regenerative microenvironment of CSTDs in vivo

**Fig. 6:**
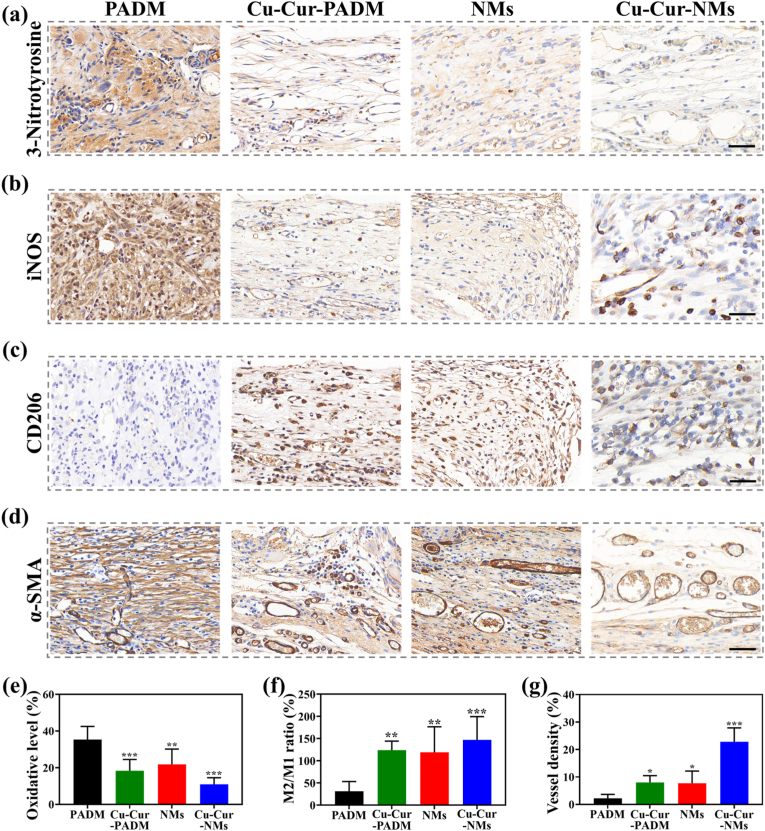
a) 3-Nitrotyrosine IHC staining of samples treated by PADM, Cu-Cur-PADM, NMs, and Cu-Cur-NMs, scale bar: 40 μm. b) iNOS IHC staining of samples from different groups, scale bar: 40 μm. c) CD206 IHC staining of samples from different groups, scale bar: 40 μm. d) α-SMA IHC staining of samples from different groups, scale bar: 40 μm. e) Quantification of oxidative level based on the images in (a). f) Quantification of M2/M1 macrophages ratio based on the images in (b) and (c). g) Quantification of vessel density based on the images in (d). Error bars represented standard deviations. ∗*p* < 0.05, ∗∗*p* < 0.01, ∗∗∗*p* < 0.001 versus PADM group.

As mentioned above, augmented oxidative stress, prolonged inflammatory response, and insufficient angiogenesis in the regenerative microenvironment delay the repair process of CSTDs [7,8]. Whether the therapeutic benefits of Cu-Cur-NMs in CSTDs management were associated with their abilities to reverse these negative aspects in regenerative microenvironment was further investigated in vivo. First, the IHC staining against 3-Nitrotyrosine (a biomarker for free radicals modified proteins) was performed to evaluate the level of oxidative stress in different groups. As shown in Fig. 6a, the extensive expression of 3-Nitrotyrosine in PADM-treated samples indicated that the over-production of free radicals in CSTDs augmented the oxidative stress in regenerative microenvironment. In contrast, the other three treatment groups all had a reduced expression of 3-Nitrotyrosine, and not surprisingly, Cu-Cur-NMs-treated samples exhibited the lowest expression (Fig. 6e). These results demonstrated that the in-vitro effects of Cu-Cur-NMs on scavenging free radicals were retained after being implanted in vivo, and successfully reshaped the oxidative microenvironment of CSTDs. Second, the populations of M1 pro-inflammatory and M2 anti-inflammatory macrophages were counted to evaluate the level of inflammatory response in different groups. As shown in Fig. 6b–c, a wide distribution of iNOS^+^ M1 macrophages was found in PADM-treated samples, along with a scattered distribution of CD206^+^ M2 macrophages. This observation suggested that the phenotypic transition of macrophages often failed in CSTDs, leading to the repair process trapped into the persistent inflammation. In contrast, the distribution of iNOS^+^ M1 macrophages was decreased in both Cu-Cur-PADM and NMs treatments, along with an increased distribution of CD206^+^ M2 macrophages. Furthermore, Cu-Cur-NMs-treated samples exhibited the highest ratio of M2/M1 macrophages among all groups, demonstrating that the in-vitro effects of Cu-Cur-NMs on reprogramming macrophages were retained after being implanted in vivo, and successfully reshaped the inflammatory microenvironment of CSTDs (Fig. 6f). Finally, the IHC staining against α-SMA (a biomarker for blood vessel wall) was performed to evaluate the angiogenic degree in different groups. It was found that few blood vessels were formed in the PADM-treated samples, indicating that the commercial biological patches were not pro-angiogenic in nature (Fig. 6d). Although Cu-Cur-PADM and NMs treatments could accelerate the angiogenic process, the quickest formation of new blood vessels was observed in Cu-Cur-NMs-treated samples. Consistently, quantitative analysis showed that the vessel density increased from 2.22 % in PADM group, to 8.00 % in Cu-Cur-PADM group, 7.73 % in NMs group, and 22.83 % in Cu-Cur-NMs group, respectively (Fig. 6g). These results demonstrated that the in-vitro effects of Cu-Cur-NMs on regulating the angiogenic behaviors of HUVECs were retained after being implanted in vivo, and successfully reshaped the ischemic microenvironment of CSTDs.

Previous studies have thoroughly explored the pro-angiogenic effects of Cu, as well as the anti-oxidative and anti-inflammatory effects of Cur on accelerating the chronic wound healing [55,56]. This study provided a rationale for the synergistic application of these two bioactive components, after transforming them into one MPNs-based nanostructure. Compared to PADM treatment, Cu-Cur-PADM treatment could restore redox homeostasis, reduce inflammatory response, and promote blood vessel formation in the regenerative microenvironment of CSTDs. However, the repair quality of AWDs treated by Cu-Cur-PADM had not yet achieved the desired outcomes. On one hand, Cu-Cur MPNs were just sprayed on the surface of PADM, and could be easily cleared from implanted site. On the other hand, Cu-Cur MPNs were unable to penetrate deep into defected site, and all of these limitations might lead to an inefficient utilization of Cu-Cur MPNs by host organism. The design of NMs appeared as an appropriate solution to resolve the dilemma. The mechanical strength, bio-adhesive property, and inherent immunomodulatory effect made NMs a superior patch system for restoring the integrity of AWDs, as evidenced by the improved outcomes from NMs-treated samples compared to PADM treatment. In addition, NMs not only provided a hydrogel matrix for encapsulating Cu-Cur MPNs to prevent their rapid clearance, but also created microchannels in implanted site to facilitate the deep penetration of Cu-Cur MPNs. Therefore, NMs could also serve as an outstanding drug delivery system to maximize the treatment potential of Cu-Cur MPNs. Based on the above descriptions, Cu-Cur-NMs became an effective treatment strategy for CSTDs management in this study without a doubt. Compared to PADM, Cu-Cur-PADM, and NMs treatments, the Cu-Cur-NMs-treated samples exhibited a more optimized regenerative microenvironment in terms of oxidative, inflammatory, and ischemic levels, resulting in the highest repair quality of CSTDs among all groups.

Recent advances in bio-fabrication techniques have facilitated the development of new patches from naturally occurring and synthetic hydrogels and polymers, providing promising alternatives for the management of CSTDs [57,58]. For instance, Wu et al. introduced a double-layer asymmetric patch for AWDs repair, by in-situ freeze-thaw of sticky polyvinyl alcohol (PVA) solution on the loosely porous surface of small intestinal submucosa (SIS) [59]. With the degradation of SIS layer, the mechanical strength of SIS/PVA patches provided dynamic support for the changing requirements during different periods of tissue reconstruction. However, because of the inherent constraints in drug loading and release performance, SIS/PVA patches could not realize deep and efficient delivery of bioactive cargos in a controlled manner, which impeded their full potential in addressing the varied microenvironment of CSTDs. In another instance, using extrusion-based three-dimensional (3D) printing technology, Eceiza et al. developed a series of personalized patches composed of alginate and waterborne-polyurethane [60]. Taking advantages of computer-aided design programs, the surface morphologies, internal geometries, mechanical properties, and chemical characteristics of fabricated patches were all precisely tailored to adapt to the speciﬁc tissue defects and patient necessities. However, the strict requirements for 3D printing equipment, limited choices for printable ink, and complex manual operations made the 3D printing patches expensive for routine use. In contrast, considering all the synthetic components of Cu-Cur-NMs were abundant in nature, and the fabrication process was simple without any assistance of awkward equipment, our proposed patch system was cost-effective and extremely comfortable for use. For future clinical applications, the size of Cu-Cur-NMs could be readily tailored to fit the shape of defected site. And the on-demand release of Cu and Cur could be tuned according to the dynamic microenvironment of CSTDs, enabling a personalized and precise treatment.

## 4 Conclusion

In summary, an NMs-based patch system with Cu-Cur MPNs encapsulation was developed in this study to accelerate the repair process of CSTDs in a comprehensive manner. Upon implantation, the NMs-based patch system could provide an adequate mechanical support for defected site without any need of sutures. Afterwards, the encapsulated Cu-Cur MPNs could disassemble in response to acidic microenvironment of CSTDs, and release Cu and Cur to exert synergistic effects. Finally, the developed Cu-Cur-NMs could remodel the oxidative microenvironment by scavenging free radical species, remodel the inflammatory microenvironment by reprogramming macrophages, and remodel the ischemic microenvironment by promoting blood vessel formation. These advantages dramatically improved the repair quality of CSTDs, convenience of use for surgeons, and comfort of implantation for patients. In addition, the underlying molecular biological mechanisms were also investigated. It was found that the anti-inflammatory effects of Cu-Cur-NMs were related to the inhibition of NLRP3 inflammasome activation in macrophages, and the pro-angiogenic effects were related to the up-regulation of HIF-1α/VEGF signaling in endothelial cells. In closing, our proposed Cu-Cur-NMs appeared as a significant advancement toward CSTDs repair by responding and remodeling the regenerative microenvironment, and ensured a high potential for clinical translation.

## CRediT authorship contribution statement

**Chengyang Zhu:** Writing – original draft, Validation, Investigation, Data curation. **Zun Fan:** Writing – original draft, Validation, Investigation, Data curation. **Zhijie Cheng:** Validation, Investigation, Data curation. **Jun Yin:** Supervision, Funding acquisition, Formal analysis, Conceptualization. **Lei Qin:** Writing – review & editing, Supervision, Formal analysis, Conceptualization. **Xin Zhao:** Writing – review & editing, Supervision, Funding acquisition, Formal analysis, Data curation, Conceptualization.

## Declaration of competing interest

The authors declare that they have no known competing financial interests or personal relationships that could have appeared to influence the work reported in this paper.

## Data Availability

Data will be made available on request.

## References

[1] Maschalidi S., Mehrotra P., Keceli B.N., De Cleene H.K.L., Lecomte K., Van der Cruyssen R., Janssen P., Pinney J., van Loo G., Elewaut D., Massie A., Hoste E., Ravichandran K.S. (2022). Targeting SLC7A11 improves efferocytosis by dendritic cells and wound healing in diabetes. Nature.

[2] Heszlein-Lossius H., Ismail A., Al-Borno Y., Shaqqoura S., Skaik N., Al Hinnawi I., Matar M., Gilbert M. (2021). Long-term health effects after conflict-related traumatic amputation among patients in Gaza. Lancet.

[3] Nakka K., Hachmer S., Mokhtari Z., Kovac R., Bandukwala H., Bernard C., Li Y., Xie G., Liu C., Fallahi M., Megeney L.A., Gondin J., Chazaud B., Brand M., Zha X., Ge K., Dilworth F.J. (2022). JMJD3 activated hyaluronan synthesis drives muscle regeneration in an inflammatory environment. Science.

[4] Caves E., Horsley V. (2022). Reindeer light the way to scarless wound healing. Cell.

[5] Shang M., Cappellesso F., Amorim R., Serneels J., Virga F., Eelen G., Carobbio S., Rincon M.Y., Maechler P., De Bock K., Ho P.C., Sandri M., Ghesquiere B., Carmeliet P., Di Matteo M., Berardi E., Mazzone M. (2020). Macrophage-derived glutamine boosts satellite cells and muscle regeneration. Nature.

[6] Hoeffel G., Debroas G., Roger A., Rossignol R., Gouilly J., Laprie C., Chasson L., Barbon P.V., Balsamo A., Reynders A., Moqrich A., Ugolini S. (2021). Sensory neuron-derived TAFA4 promotes macrophage tissue repair functions. Nature.

[7] Lu Y.Z., Nayer B., Singh S.K., Alshoubaki Y.K., Yuan E., Park A.J., Maruyama K., Akira S., Martino M.M. (2024). CGRP sensory neurons promote tissue healing via neutrophils and macrophages. Nature.

[8] Willenborg S., Sanin D.E., Jais A., Ding X., Ulas T., Nuchel J., Popovic M., MacVicar T., Langer T., Schultze J.L., Gerbaulet A., Roers A., Pearce E.J., Bruning J.C., Trifunovic A., Eming S.A. (2021). Mitochondrial metabolism coordinates stage-specific repair processes in macrophages during wound healing. Cell Metab..

[9] Pena O.A., Martin P. (2024). Cellular and molecular mechanisms of skin wound healing. Nat. Rev. Mol. Cell Biol..

[10] Harrison O. (2020). Poised for tissue repair. Science.

[11] Rios-Diaz A.J., Cunning J.R., Talwar A.A., Christopher A., Broach R.B., Hsu J.Y., Morris J.B., Fischer J.P. (2022). Reoperation through a prosthetic-reinforced abdominal wall and its association with postoperative outcomes and longitudinal health care utilization. JAMA Surg.

[12] Su J., Liu B., He H., Ma C., Wei B., Li M., Li J., Wang F., Sun J., Liu K., Zhang H. (2022). Engineering high strength and super-toughness of unfolded structural proteins and their extraordinary anti-adhesion performance for abdominal hernia repair. Adv. Mater..

[13] Zhang X., Chen X., Hong H., Hu R., Liu J., Liu C. (2021). Decellularized extracellular matrix scaffolds: recent trends and emerging strategies in tissue engineering, Bioact. Mater.

[14] Shao Z., Yin T., Jiang J., He Y., Xiang T., Zhou S. (2022). Wound microenvironment self-adaptive hydrogel with efficient angiogenesis for promoting diabetic wound healing, Bio. Mater.

[15] Zhang X., Gan J., Fan L., Luo Z., Zhao Y. (2023). Bioinspired adaptable indwelling microneedles for treatment of diabetic ulcers. Adv. Mater..

[16] Adigweme I., Yisa M., Ooko M., Akpalu E., Bruce A., Donkor S., Jarju L.B., Danso B., Mendy A., Jeffries D., Segonds-Pichon A., Njie A., Crooke S., El-Badry E., Johnstone H., Royals M., Goodson J.L., Prausnitz M.R., McAllister D.V., Rota P.A., Henry S., Clarke E. (2024). A measles and rubella vaccine microneedle patch in the Gambia: a phase 1/2, double-blind, double-dummy, randomised, active-controlled, age de-escalation trial. Lancet.

[17] You J., Yang C., Han J., Wang H., Zhang W., Zhang Y., Lu Z., Wang S., Cai R., Li H., Yu J., Gao J., Zhang Y., Gu Z. (2023). Ultrarapid-acting microneedles for immediate delivery of biotherapeutics. Adv. Mater..

[18] Shan J., Wu X., Che J., Gan J., Zhao Y. (2024). Reactive microneedle patches with antibacterial and dead bacteria-trapping abilities for skin infection treatment. Adv. Sci..

[19] Zhang X., Chen G., Wang Y., Fan L., Zhao Y. (2022). Arrowhead composite microneedle patches with anisotropic surface adhesion for preventing intrauterine adhesions. Adv. Sci..

[20] Howard R., Thumma J., Ehlers A., Englesbe M., Dimick J., Telem D. (2022). Reoperation for recurrence up to 10 years after hernia repair. JAMA.

[21] Wang Z., Fu R., Han X., Wen D., Wu Y., Li S., Gu Z. (2022). Shrinking fabrication of a glucose-responsive glucagon microneedle patch. Adv. Sci..

[22] Liu Y., Zhou M., Sun J., Yao E., Xu J., Yang G., Wu X., Xu L., Du J., Jiang X. (2024). Programmed BRD9 degradation and hedgehog signaling activation via silk-based core-shell microneedles promote diabetic wound healing. Adv. Sci..

[23] Kim T., Kim B.J., Bonacchini G.E., Ostrovsky-Snider N.A., Omenetto F.G. (2024). Silk fibroin as a surfactant for water-based nanofabrication. Nat. Nanotechnol..

[24] Xu L., Zhang Z., Jorgensen A.M., Yang Y., Jin Q., Zhang G., Cao G., Fu Y., Zhao W., Ju J., Hou R. (2023). Bioprinting a skin patch with dual-crosslinked gelatin (GelMA) and silk fibroin (SilMA): an approach to accelerating cutaneous wound healing. Mater. Today Bio..

[25] Fan Z., Zhu C., Yin J., Qin L., Zhao X. (2023). Pitaya-inspired microcarrier/hydrogel composite for chronic wound healing: one-pot preparation, tailorable structures, and versatile functionalization. Chem. Eng. J..

[26] Zhou Z., Deng T., Tao M., Lin L., Sun L., Song X., Gao D., Li J., Wang Z., Wang X., L J., Jiang Z., Lou L., Yang L., Wu M. (2023). Snail-inspired AFG/GelMA hydrogel accelerates diabetic wound healing via inflammatory cytokines suppression and macrophage polarization. Biomaterials.

[27] Wang T., Dai B., Zhu Y., Ji M., Yang P., Zhang J., Liu W., Miao Y., Liu Y., Wang S., Sun J. (2024). Gene therapy for inflammatory cascade in intrauterine injury with engineered extracellular vesicles hybrid snail mucus-enhanced adhesive hydrogels. Adv. Sci..

[28] Ajisafe V.A., Raichur A.M. (2024). Snail mucus-enhanced adhesion of human chondrocytes on 3D porous agarose scaffolds. ACS Appl. Mater. Interfaces.

[29] Singh S.K., Singh R. (2022). Nanotherapy: targeting the tumour microenvironment. Nat. Rev. Cancer.

[30] Kudruk S., Forsyth C.M., Dion M.Z., Hedlund Orbeck J.K., Luo J., Klein R.S., Kim A.H., Heimberger A.B., Mirkin C.A., Stegh A.H., Artzi N. (2024). Multimodal neuro-nanotechnology: challenging the existing paradigm in glioblastoma therapy. Pro. Natl. Acad. Sci. USA.

[31] Mahmud M.M., Pandey N., Winkles J.A., Woodworth G.F., Kim A.J. (2024). Toward the scale-up production of polymeric nanotherapeutics for cancer clinical trials. Nano Today.

[32] Guo Y., Sun Q., Wu F.G., Dai Y., Chen X. (2021). Polyphenol-containing nanoparticles: synthesis, properties, and therapeutic delivery. Adv. Mater..

[33] Yu R., Chen H., He J., Zhang Z., Zhou J., Zheng Q., Fu Z., Lu C., Lin Z., Caruso F., Zhang X. (2024). Engineering antimicrobial metal-phenolic network nanoparticles with high biocompatibility for wound healing. Adv. Mater..

[34] Shi S., Han Y., Feng J., Shi J., Liu X., Fu B., Wang J., Zhang W., Duan J. (2024). Microenvironment-triggered cascade metal-polyphenolic nanozyme for ROS/NO synergistic hyperglycemic wound healing. Redox Biol..

[35] Gurnari C., Rogers H.J. (2021). Copper deficiency. N. Engl. J. Med..

[36] Kingwell K. (2023). Copper clampdown alleviates inflammation. Nat. Rev. Drug Discov..

[37] Patteson J.B., Putz A.T., Tao L., Simke W.C., Bryant L.H., Britt R.D., Li B. (2021). Biosynthesis of fluopsin C, A copper-containing antibiotic from pseudomonas aeruginosa. Science.

[38] Gong Y., Wang P., Cao R., Wu J., Ji H., Wang M., Hu C., Huang P., Wang X. (2023). Exudate absorbing and antimicrobial hydrogel integrated with multifunctional curcumin-loaded magnesium polyphenol network for facilitating burn wound healing. ACS Nano.

[39] Ruiz de Porras V., Layos L., Martinez-Balibrea E. (2021). Curcumin: a therapeutic strategy for colorectal cancer?. Semin. Cancer Biol..

[40] Wang J., Li Y., Nie G. (2021). Multifunctional biomolecule nanostructures for cancer therapy. Nat. Rev. Mater..

[41] Chen G., Wang F., Zhang X., Shang Y., Zhao Y. (2023). Living microecological hydrogels for wound healing. Sci. Adv..

[42] Gutierrez A.M., Frazar E.M., X Klaus M.V., Paul P., Hilt J.Z. (2022). Hydrogels and hydrogel nanocomposites: enhancing healthcare through human and environmental treatment. Adv. Healthcare Mater..

[43] Liang K., Ding C., Li J., Yao X., Yu J., Wu H., Chen L., Zhang M. (2024). A review of advanced abdominal wall hernia patch materials. Adv. Healthcare Mater..

[44] Qi X., Cai E., Xiang Y., Zhang C., Ge X., Wang J., Lan Y., Xu H., Hu R., Shen J. (2023). An immunomodulatory hydrogel by hyperthermia-assisted self-cascade glucose depletion and ROS scavenging for diabetic foot ulcer wound therapeutics. Adv. Mater..

[45] Eming S.A., Murray P.J., Pearce E.J. (2021). Metabolic orchestration of the wound healing response. Cell Metab..

[46] Rose-John S., Jenkins B.J., Garbers C., Moll J.M., Scheller J. (2023). Targeting IL-6 trans-signalling: past, present and future prospects. Nat. Rev. Immunol..

[47] York A.G., Skadow M.H., Oh J., Qu R., Zhou Q.D., Hsieh W.Y., Mowel W.K., Brewer J.R., Kaffe E., Williams K.J., Kluger Y., Smale S.T., Crawford J.M., Bensinger S.J., Flavell R.A. (2024). IL-10 constrains sphingolipid metabolism to limit inflammation. Nature.

[48] Zhao X., Fan Z., Zhu C., Zhang W., Qin L. (2023). Melanin inspired microcapsules delivering immune metabolites for hepatic fibrosis management, Mater. Today Bio..

[49] Gangopadhyay A., Devi S., Tenguria S., Carriere J., Nguyen H., Jager E., Khatri H., Chu L.H., Ratsimandresy R.A., Dorfleutner A., Stehlik C. (2022). NLRP3 licenses NLRP11 for inflammasome activation in human macrophages. Nat. Immunol..

[50] Liu T., Wang L., Liang P., Wang X., Liu Y., Cai J., She Y., Wang D., Wang Z., Guo Z., Bates S., Xia X., Huang J., Cui J. (2021). USP19 suppresses inflammation and promotes M2-like macrophage polarization by manipulating NLRP3 function via autophagy. Cell. Mol. Immunol..

[51] Kratofil R.M., Shim H.B., Shim R., Lee W.Y., Labit E., Sinha S., Keenan C.M., Surewaard B.G.J., Noh J.Y., Sun Y., Sharkey K.A., Mack M., Biernaskie J., Deniset J.F., Kubes P. (2022). A monocyte-leptin-angiogenesis pathway critical for repair post-infection. Nature.

[52] Li G., Ko C.N., Li D., Yang C., Wang W., Yang G.J., Di Primo C., Wong V.K.W., Xiang Y., Lin L., Ma D.L., Leung C.H. (2021). A small molecule HIF-1α stabilizer that accelerates diabetic wound healing. Nat. Commun..

[53] Salvo J., Sandoval C. (2022). Role of copper nanoparticles in wound healing for chronic wounds: literature review. Burns Trauma.

[54] Zhao X., Fan Z., Zhang W., Huang Q., Yin J., Qin L. (2023). Hierarchically hollow microcapsules with antibacterial and angiogenic properties for abdominal wall defects treatment. Appl. Mater. Today.

[55] Liu M., Huang L., Xu X., Wei X., Yang X., Li X., Wang B., Xu Y., Li L., Yang Z. (2022). Copper doped carbon dots for addressing bacterial biofilm formation, wound infection, and tooth staining. ACS Nano.

[56] Chen B., Liang Y., Zhang J., Bai L., Xu M., Han Q., Han X., Xiu J., Li M., Zhou X., Guo B., Yin Z. (2021). Synergistic enhancement of tendon-to-bone healing via anti-inflammatory and pro-differentiation effects caused by sustained release of Mg2+/curcumin from injectable self-healing hydrogels. Theranostics.

[57] Jia B., Huang H., Dong Z., Ren X., Lu Y., Wang W., Zhou S., Zhao X., Guo B. (2024). Degradable biomedical elastomers: paving the future of tissue repair and regenerative medicine. Chem. Soc. Rev..

[58] Golebiowska A.A., Intravaia J.T., Sathe V.M., Kumbar S.G., Nukavarapu S.P. (2023). Decellularized extracellular matrix biomaterials for regenerative therapies: advances, challenges and clinical prospects. Bioact. Mater..

[59] Tang F., Miao D., Huang R., Zheng B., Yu Y., Ma P., Peng B., Li Y., Wang H., Wu D. (2024). Double-layer asymmetric porous mesh with dynamic mechanical support properties enables efficient single-stage repair of contaminated abdominal wall defect. Adv. Mater..

[60] Olmos-Juste R., Olza S., Gabilondo N., Eceiza A. (2022). Tailor-made 3D printed meshes of alginate-waterborne polyurethane as suitable implants for hernia repair. Macromol. Biosci..

