# Supplementary material for: Nature-derived microneedles with metal-polyphenolic networks encapsulation for chronic soft tissue defects repair: Responding and remodeling the regenerative microenvironment
Source: Mater Today Bio. 2025 Feb 1;31:101539. doi: 10.1016/j.mtbio.2025.101539 (PMC11869007; doi:10.1016/j.mtbio.2025.101539)
Supplement: Multimedia component 1 [file mmc1.docx]

Supplementary Material

**Nature-derived microneedles with metal-polyphenolic networks encapsulation for chronic soft tissue defects repair: Responding and remodeling the regenerative microenvironment**

Chengyang Zhu, Zun Fan, Zhijie Cheng, Jun Yin ^*^, Lei Qin ^*^, Xin Zhao ^*^

Department of General Surgery, the First Affiliated Hospital of Soochow University, Suzhou 215006, PR China.

^*^ Corresponding authors

E-mail addresses: zx15152881615@163.com (X. Zhao), doctorqinlei@163.com (L. Qin), yinjunsun@163.com (J. Yin).

**Keywords**: microneedles, metal-polyphenolic networks, drug delivery, chronic soft tissue defects, regenerative microenvironment


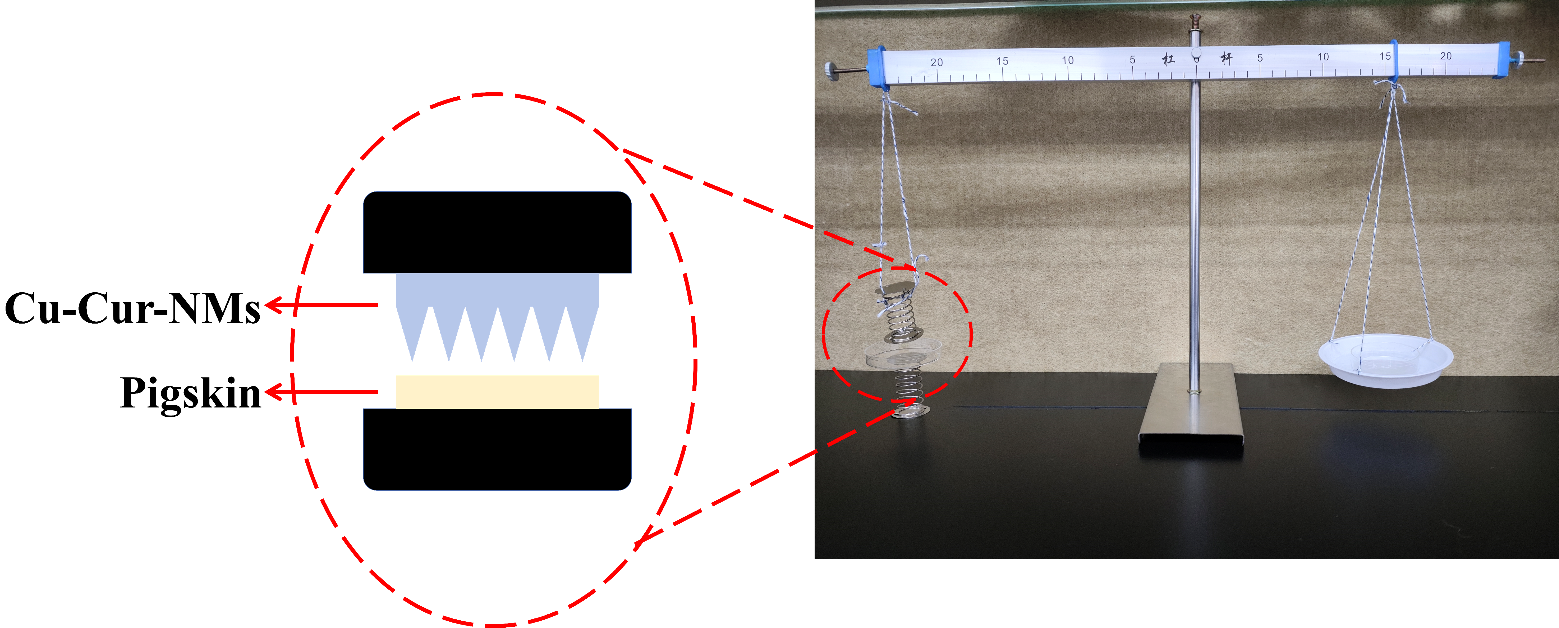


**Fig. S1**. Schematic illustration for the detection of adhesive force between Cu-Cur-NMs and pigskin.


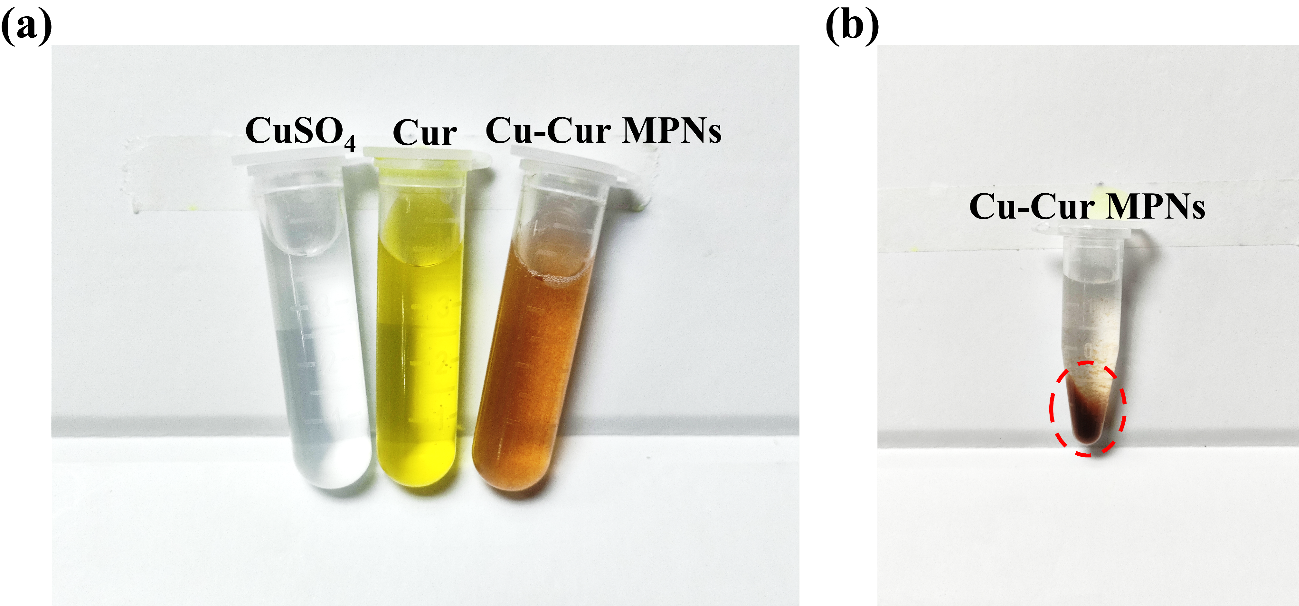


**Fig. S2**. a) Representative photograph of CuSO_4_, Cur, and Cu-Cur MPNs solutions. b) Representative photograph of collected Cu-Cur MPNs after centrifugation.


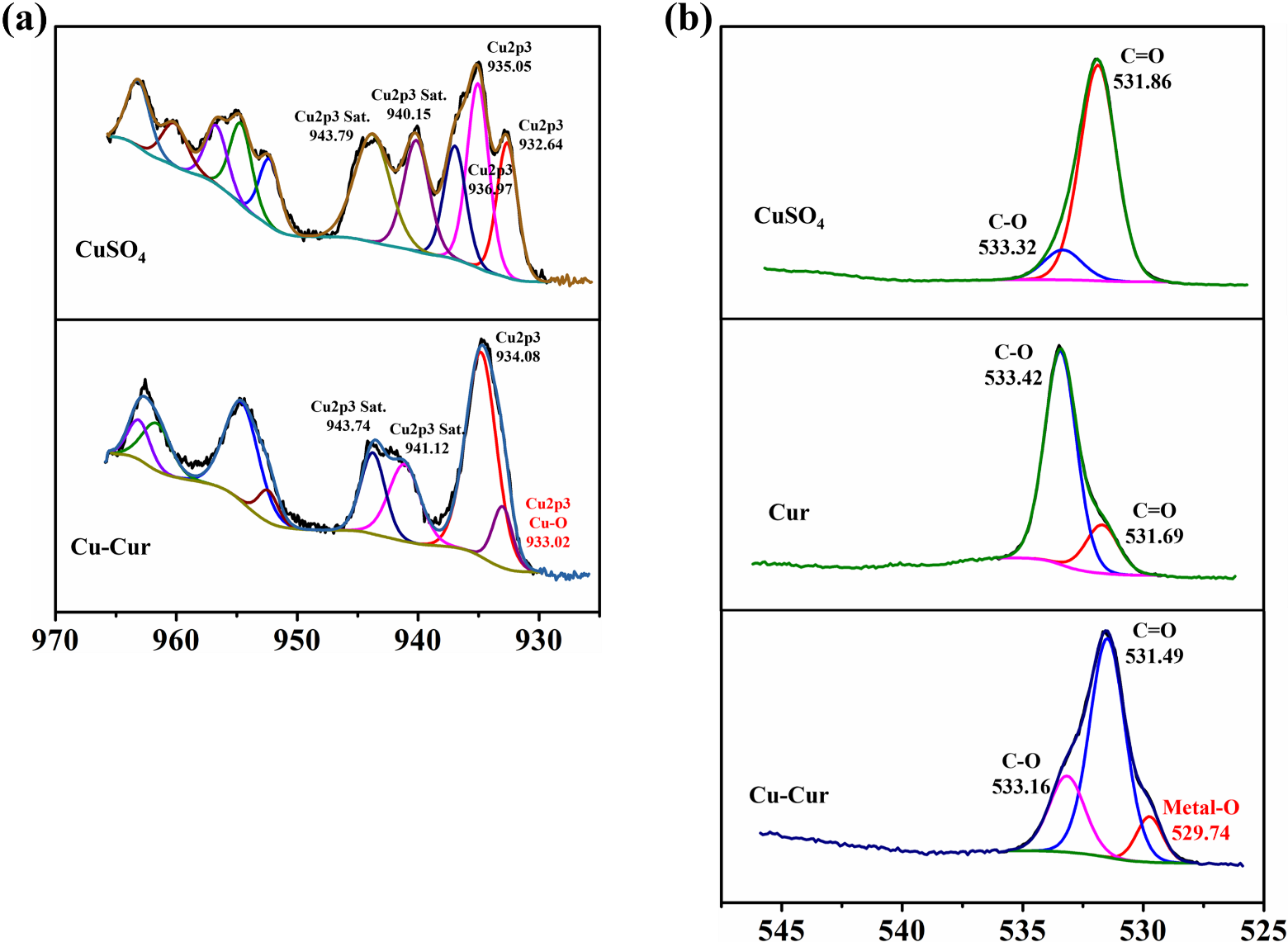


**Fig. S3**. a) High-resolution Cu2p XPS spectra of CuSO_4_ and Cu-Cur MPNs. b) High-resolution O1s XPS spectra of CuSO_4_, Cur, and Cu-Cur MPNs.


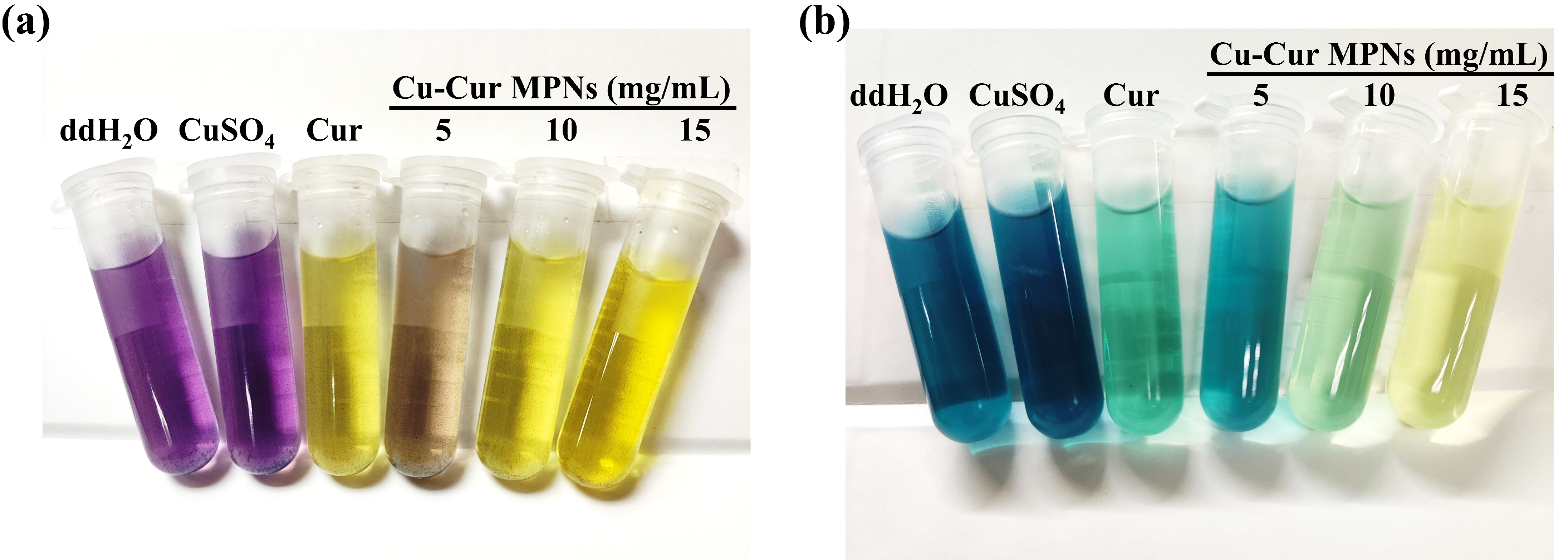


**Fig. S4**. a) Representative photograph of DPPH test solutions after being incubated with ddH_2_O, CuSO_4_, Cur, and 5-15 mg/mL Cu-Cur MPNs. b) Representative photograph of ABTS test solutions after being incubated with ddH_2_O, CuSO_4_, Cur, and 5-15 mg/mL Cu-Cur MPNs.


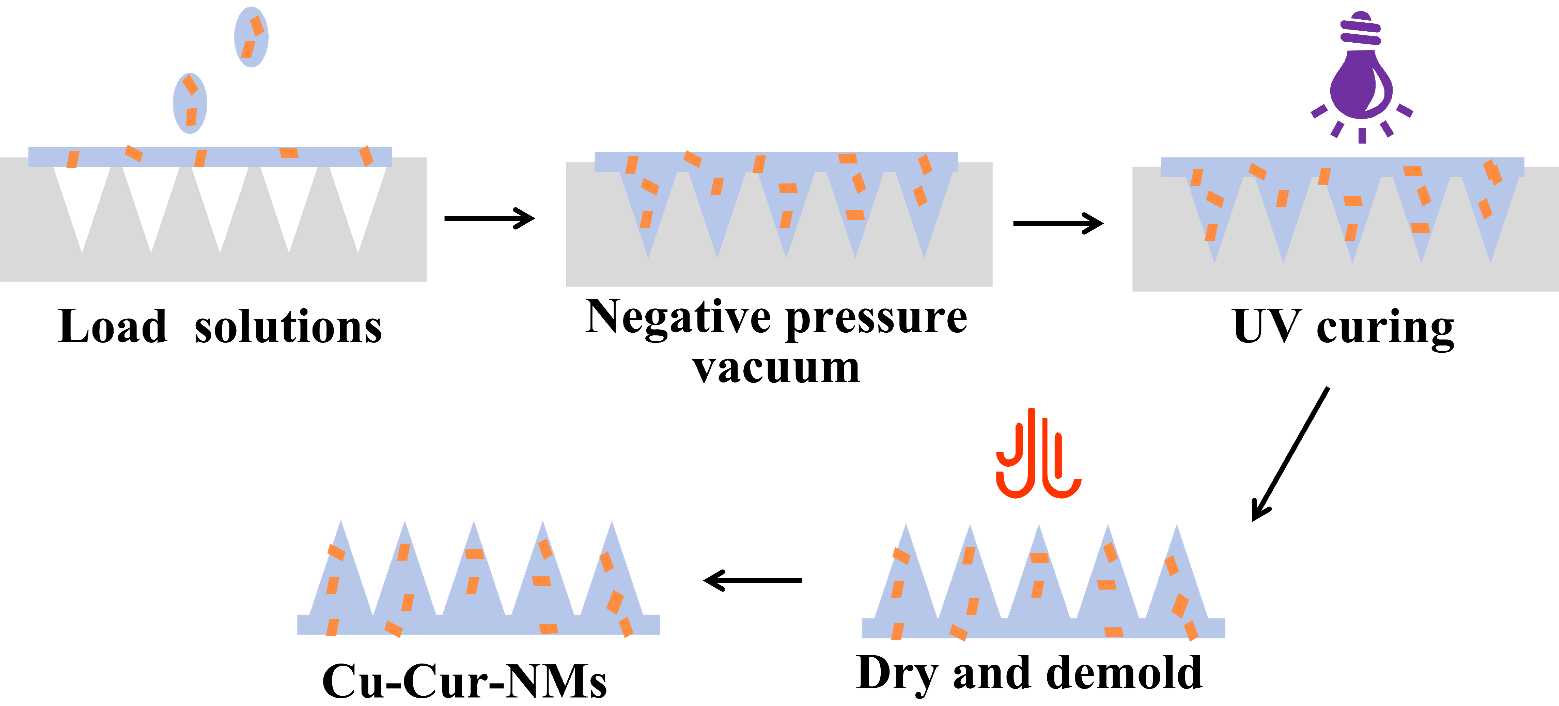


**Fig. S5**. Schematic illustration for the preparation process of Cu-Cur-NMs.


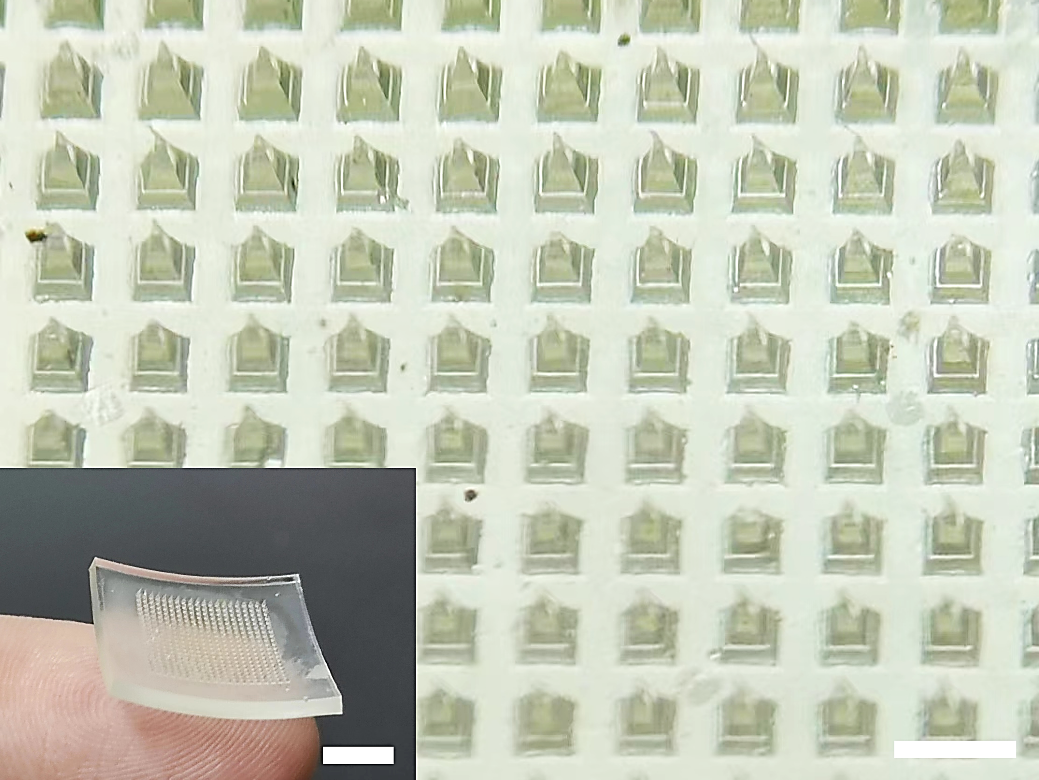


**Fig. S6**. Representative photograph of intact NMs and amplified microneedle arrays under optical microscopy, scale bar: 800 µm (5 mm in insert).


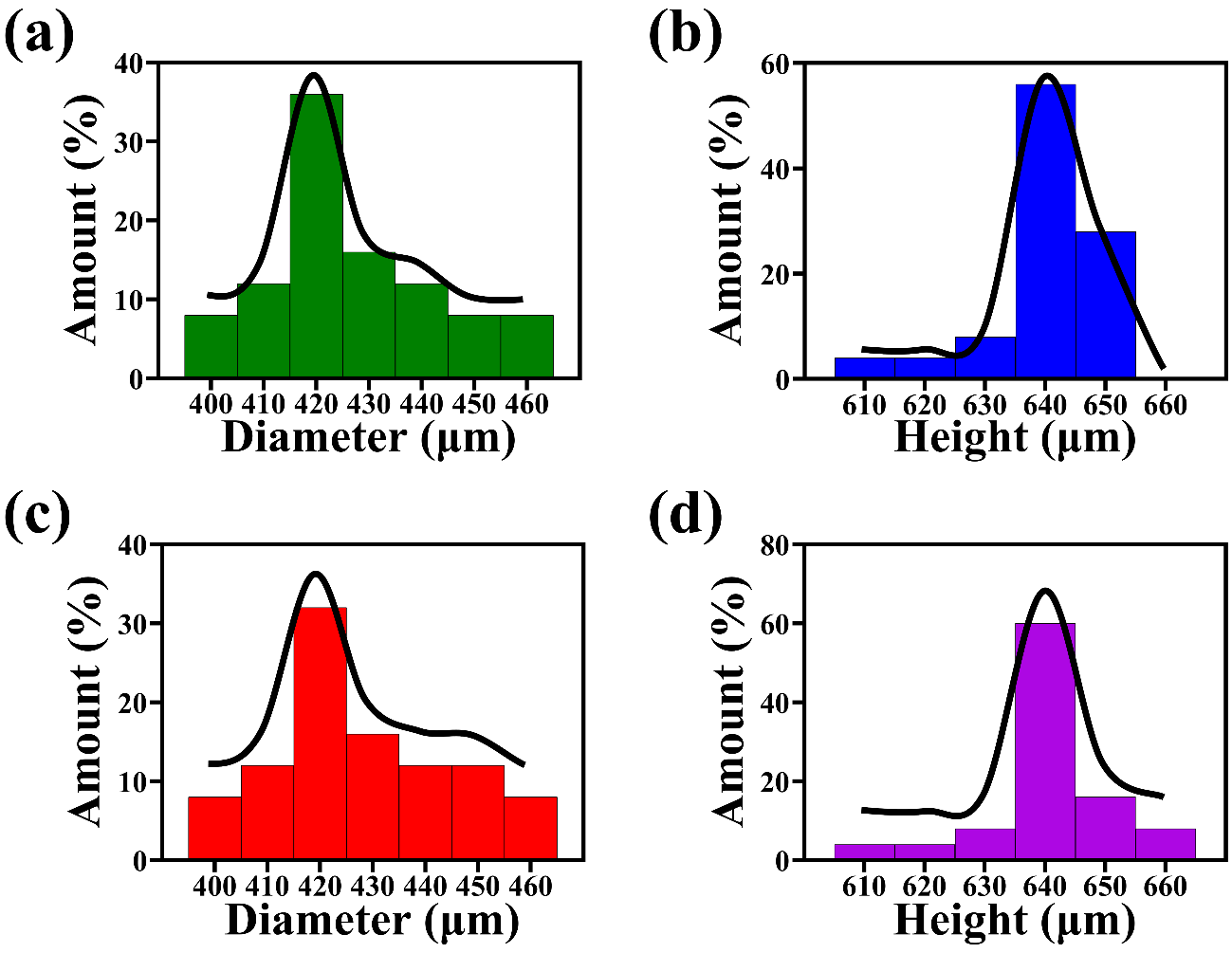


**Fig. S7**. a) Statistical distribution for the diameters of microneedle arrays from NMs. b) Statistical distribution for the heights of microneedle arrays from NMs. c) Statistical distribution for the diameters of microneedle arrays from Cu-Cur-NMs. d) Statistical distribution for the heights of microneedle arrays from Cu-Cur-NMs.


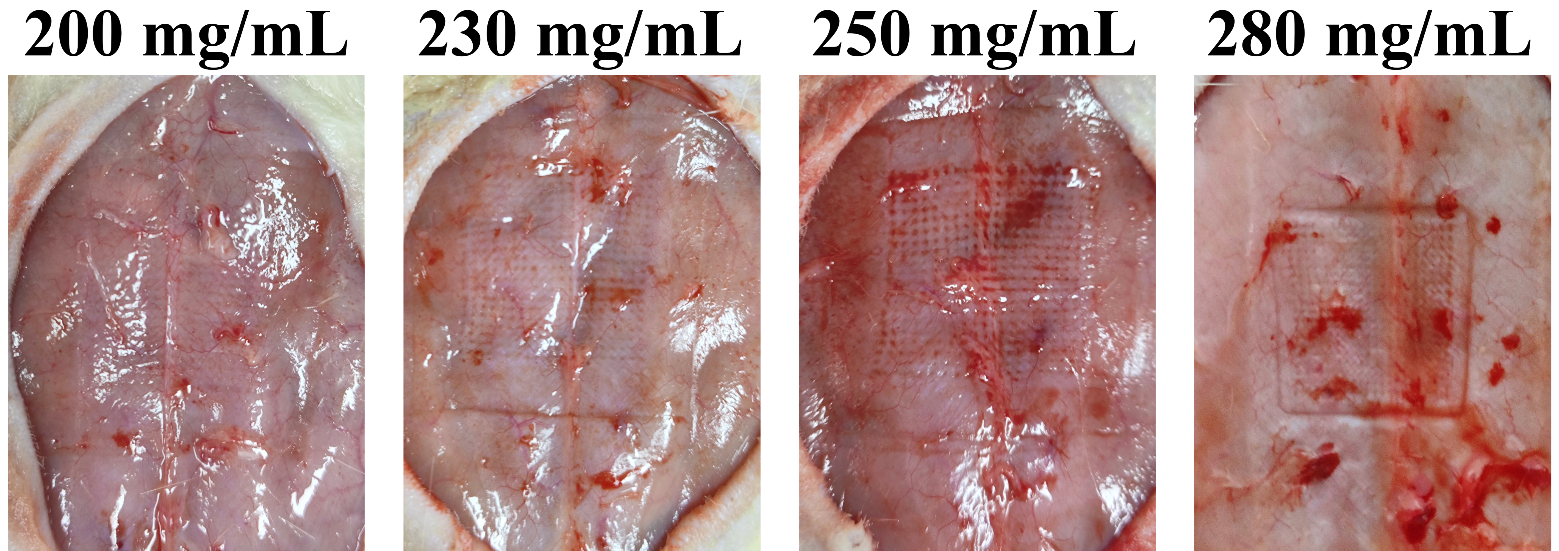


**Fig. S8**. Microchannels created on the abdominal wall of rats after removal of the implanted Cu-Cur-NMs with different concentrations of SilMA.


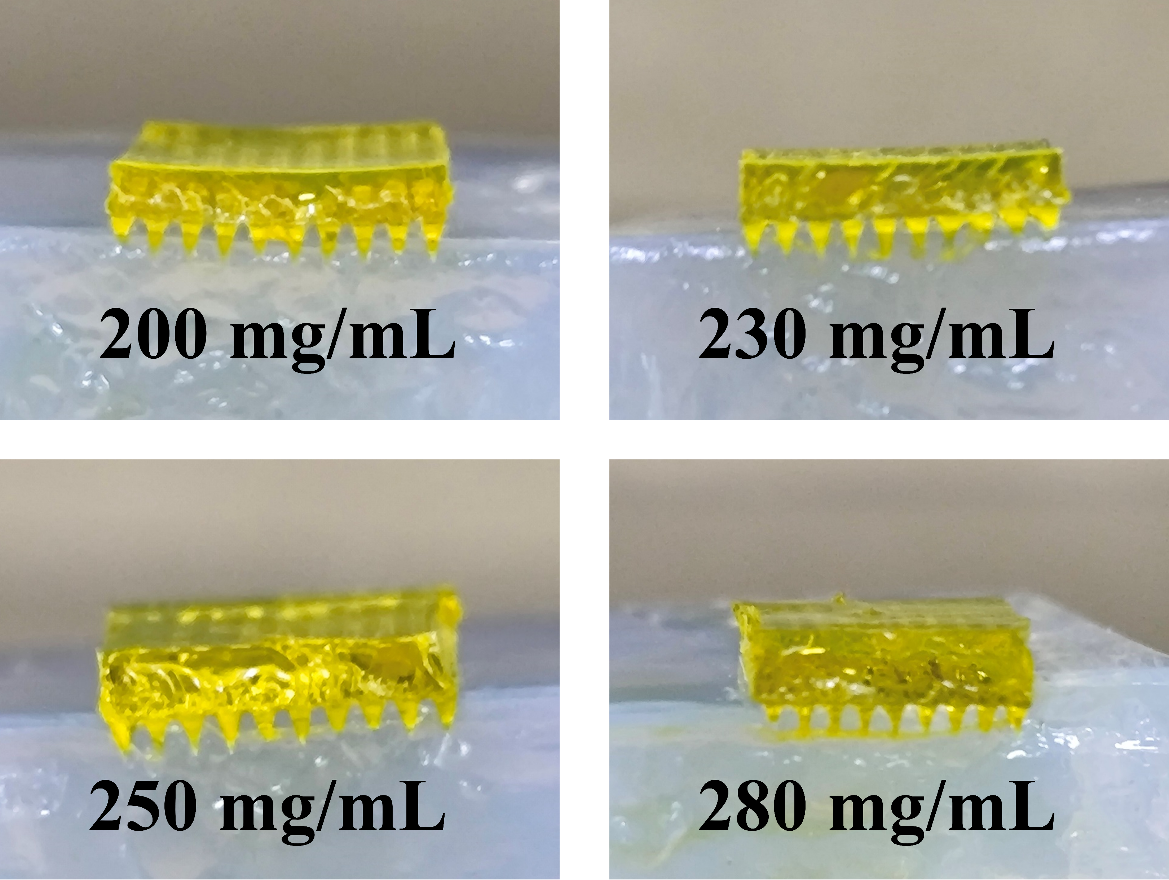


**Fig. S9**. Cu-Cur-NMs with different concentrations of SilMA were pressed onto agarose block to measure the penetration depth.


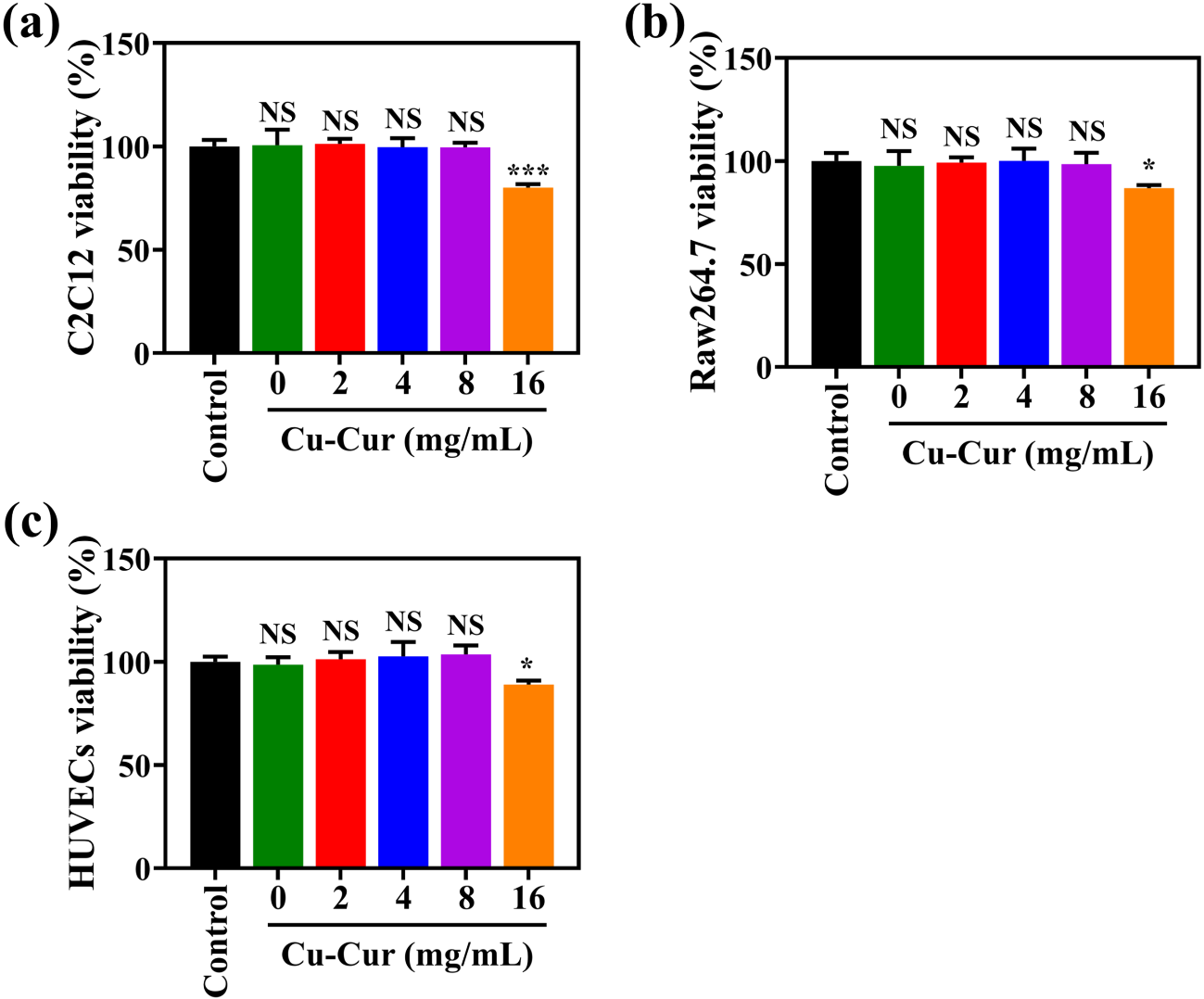


**Fig. S10**. In-vitro cell viabilities of C2C12 myoblasts, Raw264.7 macrophages, and HUVECs incubated with Cu-Cur-NMs containing various concentrations of Cu-Cur MPNs. Error bars represented standard deviations. **p* < 0.05, ****p* < 0.001, NS not significant versus control group.


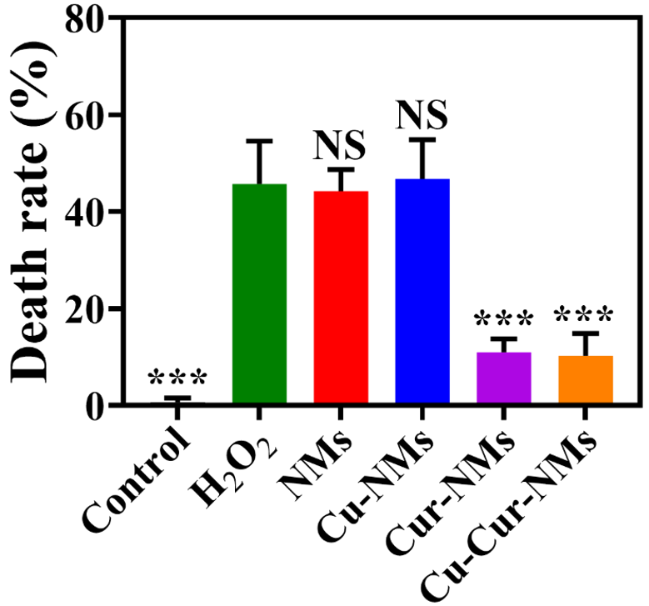


**Fig. S11**. Quantification of death rate based on the images in Fig. 3(a). Error bars represented standard deviations. ****p* < 0.001, NS not significant versus H_2_O_2_ group.


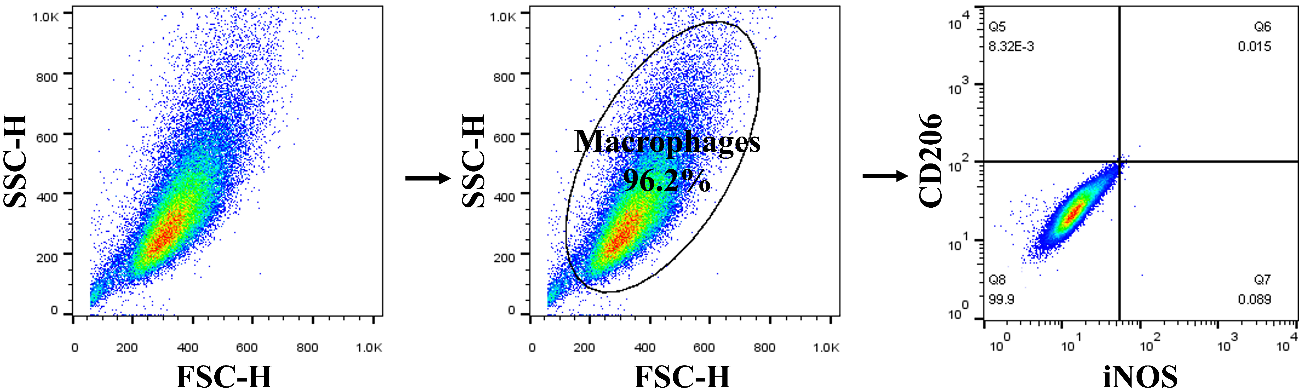


**Fig. S12**. Flow cytometry gating strategy for identifying macrophages in Fig. 3(e).


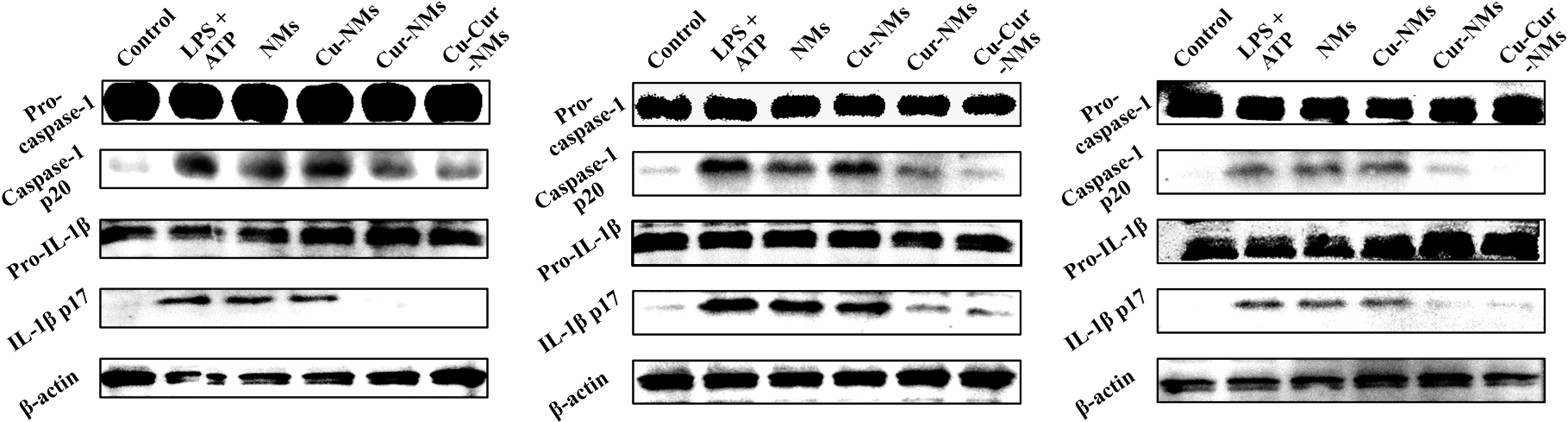


**Fig. S13**. WB analysis of caspase-1 and IL-1β activation in Raw264.7 macrophages from different groups, the experiments were repeated 3 times.


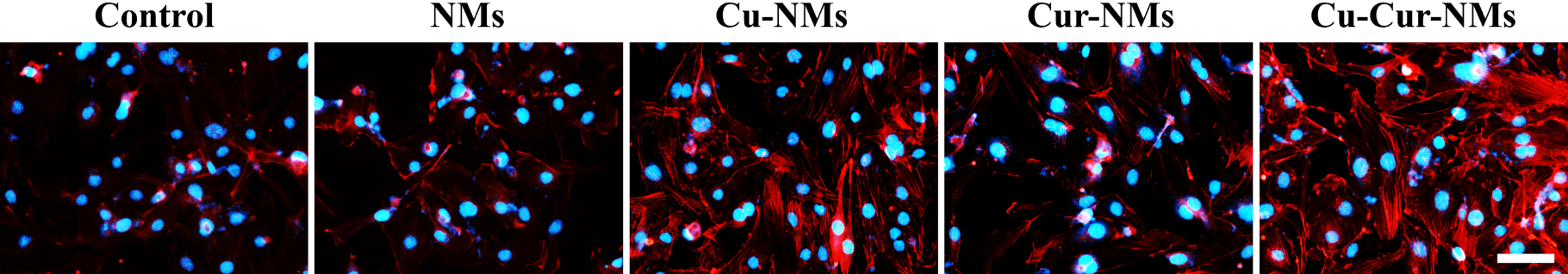


**Fig. S14**. Fluorescent images of HUVECs from different groups, after being stained with rhodamine phalloidin (red) and Hoechst 33342 (blue), scale bar: 50 μm.


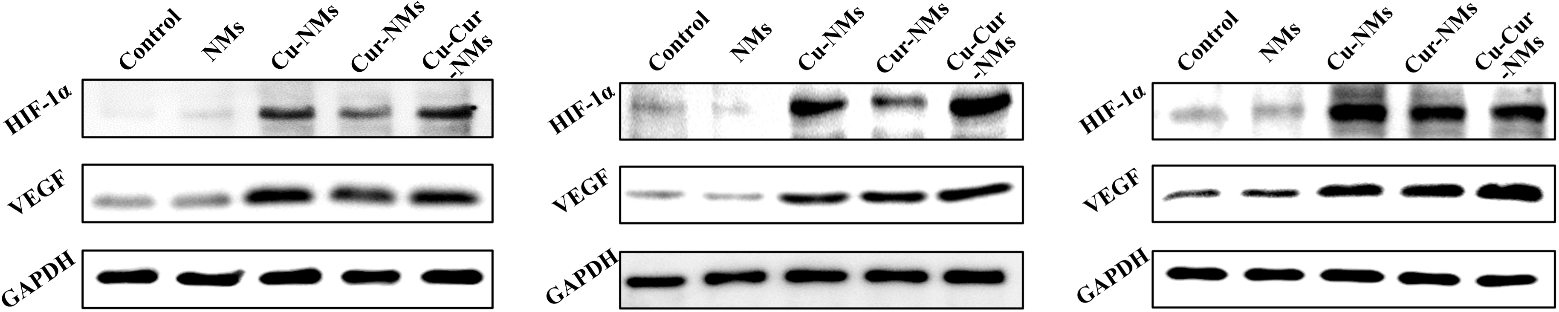


**Fig. S15**. WB analysis of HIF-1α and VEGF proteins in HUVECs from different groups, the experiments were repeated 3 times.


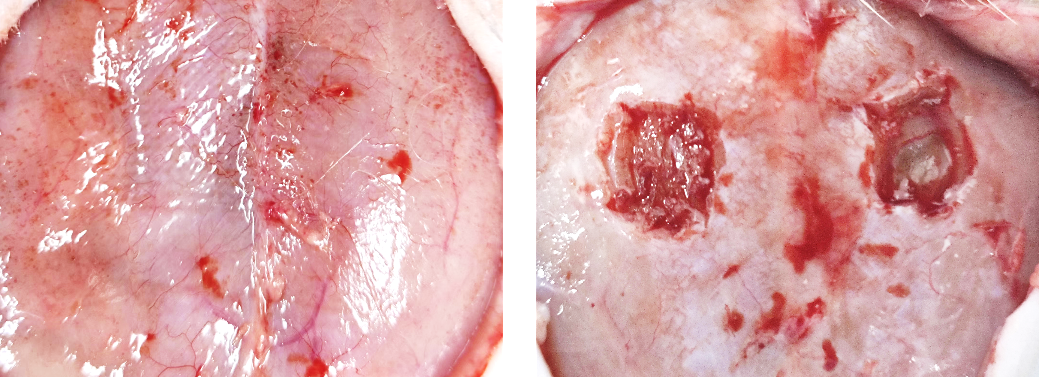


**Fig. S16**. Establishment of the bilateral 15 × 15 mm partial-thickness AWDs in diabetic rats.
